# A Residency Interview Training Program to Improve Medical Student Confidence in the Residency Interview

**DOI:** 10.15766/mep_2374-8265.10917

**Published:** 2020-07-02

**Authors:** Katelyn Donaldson, Sruthi Sakamuri, Jesse Moore, Elise N. Everett

**Affiliations:** 1 Resident, Medical University of South Carolina; 2 Resident, Pennsylvania Hospital; 3 Associate Professor, Department of Surgery, University of Vermont Medical Center; 4 Associate Professor, Department of Obstetrics, Gynecology, and Reproductive Sciences, University of Vermont Medical Center

**Keywords:** Residency Interview, Simulation, Match, Student Affairs, Admissions/Selection, Career Choice, Communication Skills, Professionalism, Self-Assessment

## Abstract

**Introduction:**

The Residency Match is becoming more competitive each year, and more than ever, medical students must prove themselves in the residency interview. Data from the 2018 National Residency Matching Program's Program Director Survey highlight the importance of the interview on Match performance. We developed a residency interview training program with the goal of preparing medical students for residency interviews, and we assessed the impact of the training on medical student confidence.

**Methods:**

Our residency interview training program includes (1) a short didactic session on the residency interview process, (2) an informational packet with commonly asked questions, (3) two 20-minute practice (mock) interviews, (4) 10 minutes of face-to-face feedback from interviewers, (5) a facilitated group debriefing, and (6) access to a participant's two videotaped interviews and a guided self-assessment. To evaluate the effectiveness of our program, we assessed student confidence with a pre- and postsurvey.

**Results:**

We have been running our residency interview training program since 2014. Over the last 5 years, 73 fourth-year medical students have participated. When polled after completing their first authentic residency interview, students felt they had more knowledge of the interview process, better preparation, and more confidence in their skills to interview successfully; they also believed that the program improved their interview performance.

**Discussion:**

Performance during the residency interview is the most important factor used by residency programs to rank applicants. Using our residency interview training program, medical students can improve their confidence prior to interviews.

## Educational Objectives

By the end of this activity, learners will be able to:

1. Describe the residency interview process.
2. Practice effective residency interviewing skills.
3. Incorporate feedback to improve their interview performance.
4. Self-assess their preparedness for residency interviews.

## Introduction

The selection process for applicants to residency programs becomes increasingly competitive each year, as there is a steadily increasing applicant pool for a relatively stable number of residency positions. The 2018 National Resident Matching Program (NRMP) had a record-breaking total of 37,103 applicants for 30,232 first-year and 2,935 second-year residency positions.^1^ For example, within the fields of obstetrics and gynecology and general surgery, applicants face increasing competition as medical students boast increasingly accomplished curricula vitae and apply to an increasingly larger number of programs to meet the competitiveness of their chosen specialty and maximize their odds of a successful Match. More specifically, for obstetrics and gynecology programs, 1,745 applicants competed for 1,336 residency positions, with an average of 1.31 applicants per position in 2018.^2^ Meanwhile, general surgery programs had 1,955 applicants for 1,319 resident slots, for an average of 1.48 applicants per position for the 2017–2018 residency application cycle.^2^ Thus, residency applicants are seeking avenues to become more competitive to attain a successful Match.

The application process consists of the standardized Electronic Residency Application Service (ERAS) application complete with United States Medical Licensing Examination step scores, medical school transcripts, a personal statement, faculty letters of recommendation, and a curriculum vitae, coupled with formal in-person interviews based on each program's selection criteria. This formal interview process arguably allows program personnel to best witness the intangible features of a student's personality and natural ability to communicate and connect with others. The NMRP surveyed program directors in all specialties across the United States for the 2017–2018 application cycle. Almost universally, program directors identified the three most important factors in ranking applicants as interactions with faculty, interpersonal skills, and interactions with house staff during the interview and program site visit.^3^ These data highlight the significance of the interview for applicant success in the Residency Match process.

Because the interview portion is critical in a program's ranking algorithm, we developed a structured residency interview training program to prepare medical students for their authentic residency interviews. Our program adds to the literature on mock interviews by being more intentional, more formalized, and more structured. The goal of the training program is to prepare medical students for the residency interview by improving knowledge of the interview process, interview skills, and self-confidence in residency interview performance. Our program provides opportunities for knowledge acquisition, skill practice, feedback, reflection, and, finally, application in the authentic residency interviews. In the absence of an ethical way to capture medical student interview performance in authentic residency interviews, we use student self-confidence after the training program as a proxy outcome measure.

## Methods

We developed a structured residency interview training program that included (1) a short didactic session on the residency interview process (Appendix A), (2) an informational packet with commonly asked questions (Appendix B), (3) two 20-minute practice (mock) interviews using a standard set of questions (Appendix C), (4) 10 minutes of face-to-face feedback using the assessment (Appendix D), (5) a facilitated debriefing (Appendix E), and (6) access to a participant's two videotaped interviews and a guided self-assessment (Appendix F). In addition, a survey (Appendix G) was completed by students before and after the interview training.

We aimed to run our residency training program near the end of September or early October in order to complete the training before authentic residency interviews began. First, a medical education coordinator identified a date when the majority of students could attend. Once the number of students participating was determined, the coordinator knew how many interviewers to recruit. The coordinator identified available interviewers and reserved the date, time, and space needed for the training program. Students submitted their ERAS application, a curriculum vitae, and a personal statement to the coordinator. The coordinator then created an interviewer packet that consisted of the following: the ERAS application, the curriculum vitae, and the personal statement for each of the students to be interviewed; a list of standardized, commonly asked interview questions (Appendix B); and the assessment form for each student (Appendix D). The coordinator also created student packets that consisted of the previous year's NRMP Match data for the student's specialty, a paper copy of the didactic slide presentation (Appendix A), and a paper copy of the informational packet with the commonly asked questions (Appendix B).

Students and interviewers were asked to arrive in professional attire. The didactic session was meant to mimic the group session given by the residency program director, except instead of talking about a specific residency program, the content was related to the residency interview process, interview skills, and interview dos and don'ts. A facilitator experienced with residency interviews such as a clerkship or program director presented this session. The session was approximately 30–40 minutes long and was a combination of didactics with guiding slides and a question-and-answer small-group segment. Students and interviewers then transitioned to the interview rooms. Five to 10 minutes were allowed for this transition. Students knocked on the door and were greeted by their interviewer and directed to a chair. Interviewers then initiated a 20-minute interview using the standard questions from Appendix C. Interviewers were given a 2-minute warning at 18 minutes, and the interview then concluded. Next, the interviewer and the student reviewed the assessment form, and the interviewer provided direct, face-to-face, immediate feedback. The feedback portion lasted 10 minutes, with a 2-minute warning given at 8 minutes. Students then transitioned to their second interview with a different interviewer, and the process repeated itself. Five to 10 minutes were allowed for this transition. All interviews were video recorded.

After the practice interviews, interviewers and students met as a large group and participated in a facilitated debriefing using the resource in Appendix E. The following day, the video recordings were released to the students via email, and students independently watched the videos and did the guided self-assessment or reflection exercise in Appendix F.

### Selection of the Facilitator and Interviewers

The facilitator and the interviewers were faculty who had experience with medical student, residency, and fellow interviews. This included faculty who held medical education leadership positions, such as a department chair, a vice-chair for education, a clerkship or program director, a clerkship or program associate director, or a fellowship director. If additional interviewers were necessary, faculty from other medical disciplines, fellows, or senior residents could be solicited.

### Selection of Students

Our sessions were done with fourth-year medical students who had submitted a residency application through ERAS. The training program occurred in late September/early October, before the authentic residency interviews began, and the students matched in March of the following year. Our training program was voluntary, not mandatory, and focused on general surgery and obstetrics and gynecology applicants due to the competitive nature of those specialties.^1^ However, we believe our program would be generalizable to any residency applicant.

### Other Resources

To deliver our training program successfully, we used a medical education coordinator and a simulation technician from our simulation center. Our didactic and debrief sessions occurred in a large debrief room in our simulation center. The interviews took place in individual rooms within the simulation center. Each room was equipped with two chairs, one for each participant, and a video recorder. The simulation technician was responsible for recording the sessions, timing the interviews and feedback sessions, and alerting participants at the 2-minute warning and the end of the sessions.

### Creation of Materials

The didactic slide presentation (Appendix A), the informational packet (Appendix B), and the list of nine questions (Appendix C) were created internally. The content for these was informed by several sources.^4^ Our internal medical education experts felt the content in these three sources was representative of the residency interview.

The interview performance assessment or evaluation was created internally, by asking local program directors for a copy of the evaluation form they used for their residency interviews. We then created the form provided in Appendix D. It used a 4-point Likert-type scale, with 1 representing a polite but unengaged student and 4 representing a student in the top fifth percentile. The furthest column on the right had a score of 0 and represented the unprofessional, arrogant student that a program would not rank.

There were several debriefing scripts in the literature. We based our debriefing script (Appendix E) on a script called Promoting Excellence and Reflective Learning in Simulation (PEARLS).^5^ This script was specifically designed for new debriefers and could be easily adapted to multiple activities. This script had four domains (Reaction, Description, Analysis, and Application) and consisted of the following five questions:

1. How is everyone feeling? Or: How did that feel? (Reaction)
2. Can someone summarize the experience? (Description)
3. What aspects of the interview went well? (Analysis)
4. What aspects of the interview were challenging? (Analysis)
5. What is one takeaway that will help you in the future? What is one thing you will change to perform better in a future interview? (Application)

Finally, the guided self-assessment or reflection (Appendix F) for students to complete while watching their recorded videos was created internally by a faculty medical educator with an interest and experience in the role of reflection in medical education.

### Description of the Appendices and Their Use in the Activity

• Appendix A: the slide presentation used in the large-group didactic session prior to the two practice interviews.
• Appendix B: the informational packet provided to students at the didactic session to be used as an ongoing resource after the training program.
• Appendix C: the 10 standardized questions provided to interviewers and answered by students during the two practice interviews.
• Appendix D: the tool used by the interviewers to evaluate student performance and provide feedback during the two feedback sessions after the interviews.
• Appendix E: questions that could be used by the training program facilitator during the large-group debriefing session occurring immediately after the two practice interviews.
• Appendix F: the guided self-assessment that the students use when watching their video-recorded interviews.
• Appendix G: the pre- and posttraining confidence survey assessing student confidence related to the residency interview.

## Results

We have been running our residency interview training program since 2014 and have had 73 students complete it. Pilot data from 29 students completing the residency interview training in 2015 demonstrated that medical students had limited prior experience with either medical school or job interviews. Likert score–based pre- and postsurveys were administered to all 29 students. Chi-square analysis of differences in mean scores between pre- and postsurveys demonstrated statistically significant increases in confidence in how to dress (pre = 4.50, post = 4.95, *p* < .001), how to introduce and present oneself (pre = 3.50, post = 4.90, *p* < .001), what questions to ask (pre = 2.50, post = 4.48, *p* < .006), and ability to perform well in an authentic residency interview (pre = 3.50, post = 4.05, *p* = .002) After students completed their first authentic residency interview, their responses regarding the merits of the training program were highest. Survey results from our pilot participants after their first authentic residency interviews are shown in the Table. In general, students found the opportunity to do multiple practice interviews and the individualized, immediate, specific, and honest feedback to be the most valuable portions of the program.

**Table.**
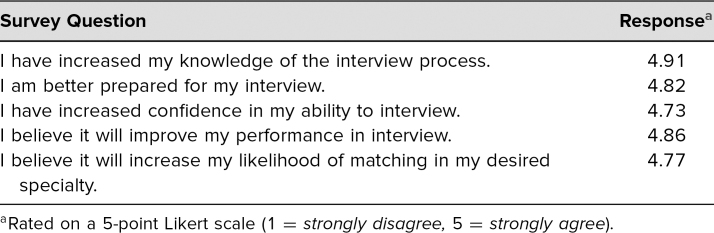
Survey of Students’ Attitudes After First Authentic Residency Interview (*n* = 29)

## Discussion

Residency interview performance contributes heavily to a candidate's overall rank, with the majority of program directors citing factors directly related to the interview day as having the greatest weight in the selection process. However, medical students are often underprepared for the gravity of the residency interview. We believe that residency interviewing is a professional activity with its own set of knowledge, skills, attitudes, and behaviors that can be taught. Using a residency interview program with the ability to practice and receive feedback, medical students can be better prepared, more confident, and perform better in the interview, potentially improving their rank by the program, with the ultimate objective of improving outcomes in the Match.

Limitations of our program include no formal training for the interviewers, no simulation by interviewers of archetypal roles such as a distracted or hostile interviewer, and a heavy reliance on faculty time and simulation center resources. In addition, the videotaped interviews were not reviewed by interviewers, and students were not guaranteed a third interview after they had had time to review the entire program and reflect on and incorporate the feedback. However, we do not feel that the failure to train interviewers or have them simulate other roles is a major flaw. We would argue that most interviewers performing residency interviews have had no training, so our program's lack of training is actually typical of what students will see in authentic residency interviews. In addition, simulating other interviewer roles is not necessary. Due to the competitive environment, most programs are trying to impress applicants, and interviewers are generally engaged, positive, and polite, making simulation unnecessary.

How much time it takes to run the program is dependent on the number of students and the number of interviewers. For example, if one has eight students and only four interviewers, then having each student do two 20-minute interviews with 10 minutes of feedback takes 2 hours. If one has eight students and eight interviewers, then it takes only 1 hour. If time is of the essence, there are numerous ways to shorten the session. First, the didactic slide presentation with the students and the facilitator on can be offered on a different date from the actual interviews. To save time, one can shorten the interview length, the feedback length, or both. If there are a lot of students and very few faculty, then recruiting others such as fourth-year students, residents, fellows, or faculty from other disciplines to be interviewers can be useful. As an example, in 2018, we ran the program with 120 rising fourth-year students. We did the didactic session with 120 students and one facilitator, then had students form groups of three to make 40 small groups. Each student in a small group took a turn as the interviewer, the interviewee, and, using a smart phone, the videographer. We had five faculty circulating the room to answer student questions. Finally, we have found other ways for students to review their interview videos and practice more interviews by asking them to seek out other mentors, advisors, or letter writers to perform these roles outside the structured training program. Students are encouraged to arrange a time to meet with their mentor, review the videos, receive additional feedback, and then practice another interview with the mentor either immediately following or at a later date and time.

The tangible benefits of a residency interview program are difficult to quantify since an applicant's success in the Match process is based on a multitude of factors. However, evidence suggests that there are no negative consequences of such preparation and that student interview performance can only be improved. To our knowledge, the current literature has not yet examined the effect of residency interview programs on medical student Match outcomes. Studies have shown improved success in the Match process among pharmacy students.^6,7^ Koenigsfeld and colleagues demonstrated a higher Match rate among pharmacy students who completed their faculty-orchestrated mock residency interview exercise as compared to the American Society of Health-System Pharmacists national rate.^6^ Therefore, given the success of mock interviews among pharmacy students, the concept of interview programs can be translated for medical school students applying to residency programs. Residency interview training programs offer an unparalleled resource for medical students’ interview process preparation, allowing the opportunity for practice and individualized feedback on one of the most critical components of the ranking process: the interview.

## Appendices

Didactic Slide Presentation.pptxInformational Packet for Students.docxQuestions for Facilitators.docxInterview Performance Evaluation Tool.docxDebriefing Script.docxGuided Self-Assessment.docxPre- and Posttraining Confidence Survey.docx
*All appendices are peer reviewed as integral parts of the Original Publication.*
