# Supplementary material for: A Residency Interview Training Program to Improve Medical Student Confidence in the Residency Interview
Source: MedEdPORTAL. 2020 Jul 2;16:10917. doi: 10.15766/mep_2374-8265.10917 (PMC7373200; doi:10.15766/mep_2374-8265.10917)
Supplement: Supplementary file 1 — Didactic Slide Presentation.pptxInformational Packet for Students.docxQuestions for Facilitators.docxInterview Performance Evaluation Tool.docxDebriefing Script.docxGuided Self-Assessment.docxPre- and Posttraining Confidence Survey.docx [file mep_2374-8265.10917-s001.zip › A. Didactic Slide Presentation.pptx]

## Slide 1
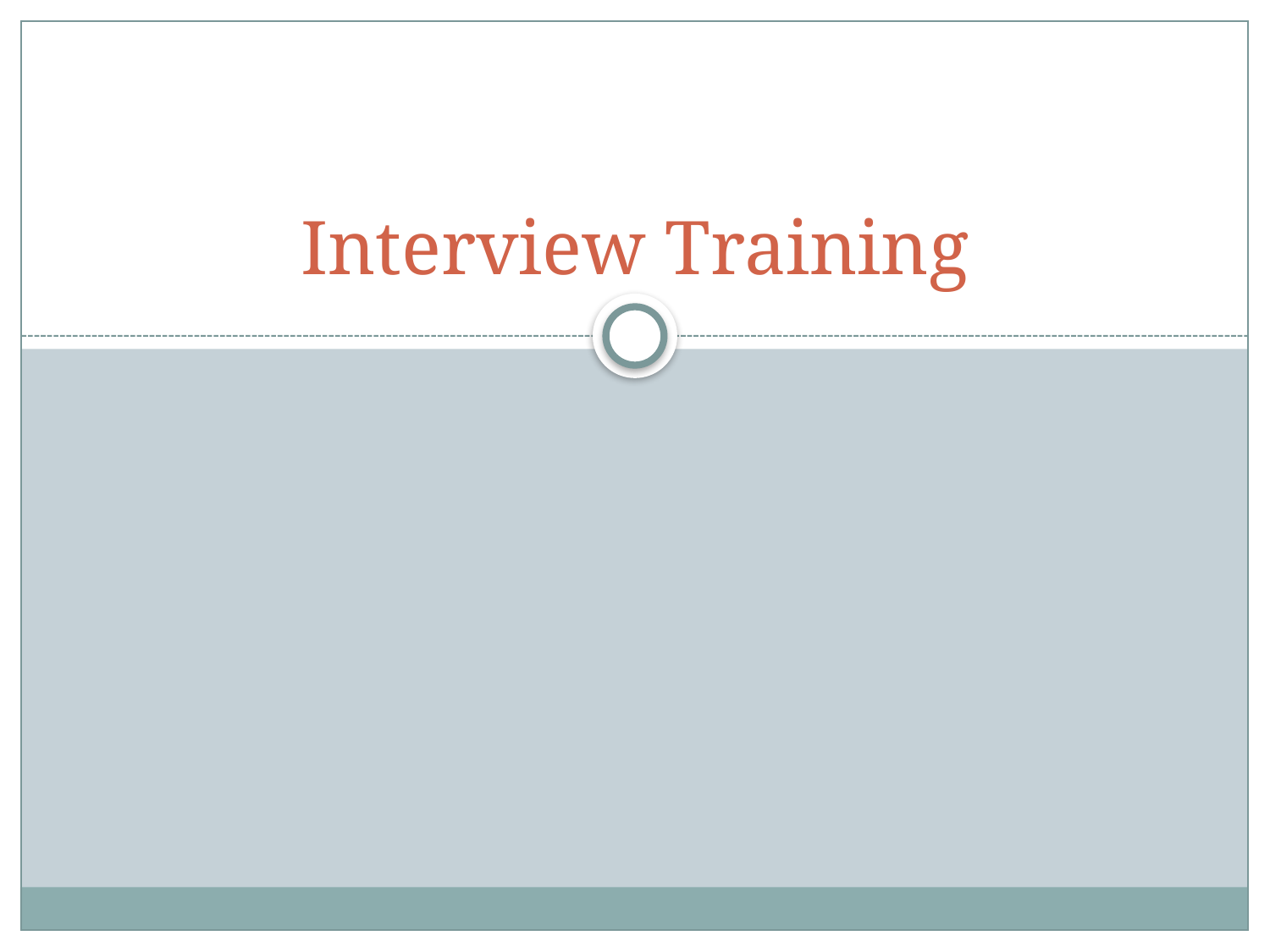

# Interview Training

## Slide 2
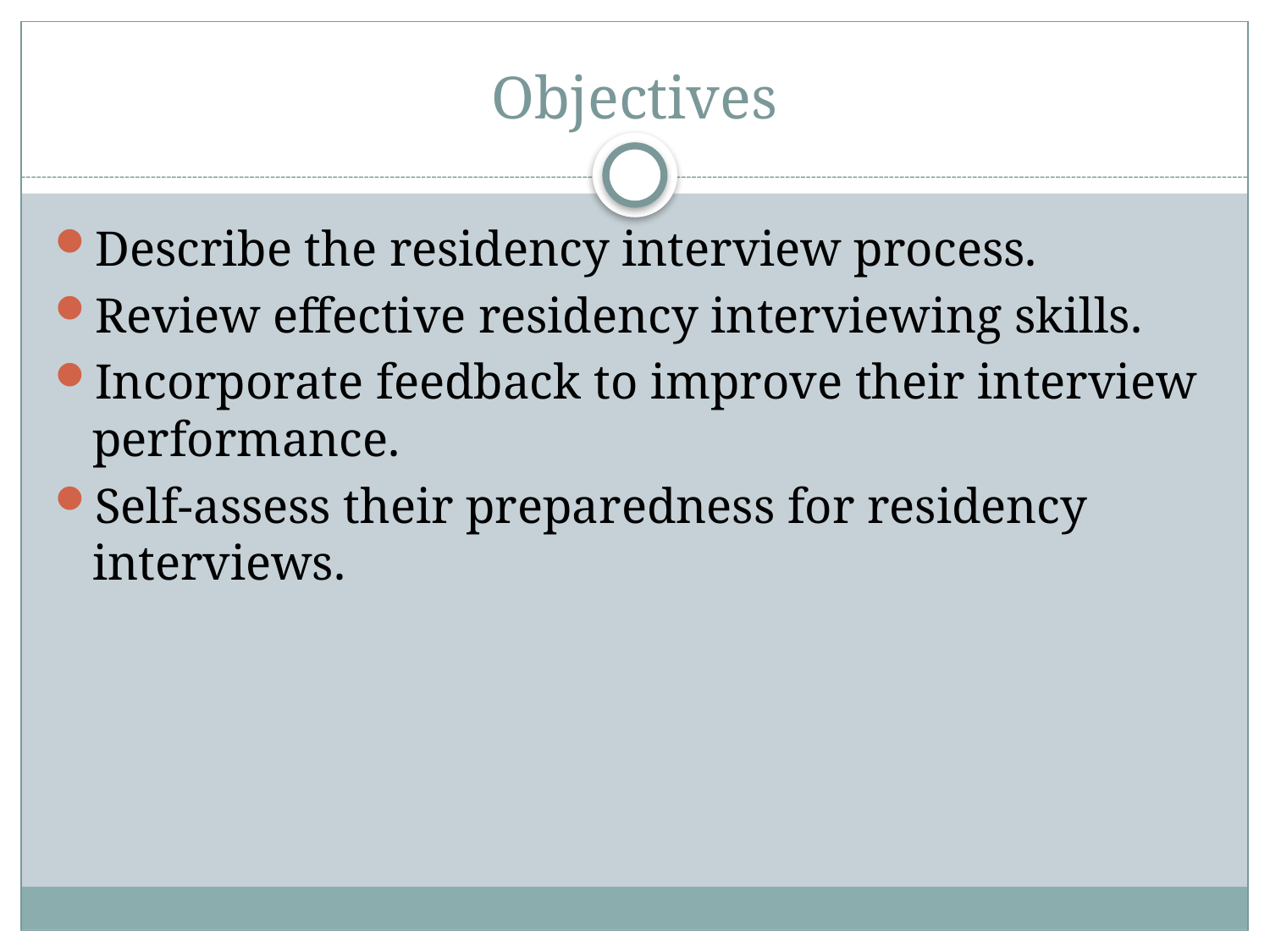

# Objectives
Describe the residency interview process.
Review effective residency interviewing skills.
Incorporate feedback to improve their interview performance.
Self-assess their preparedness for residency interviews.

## Slide 3
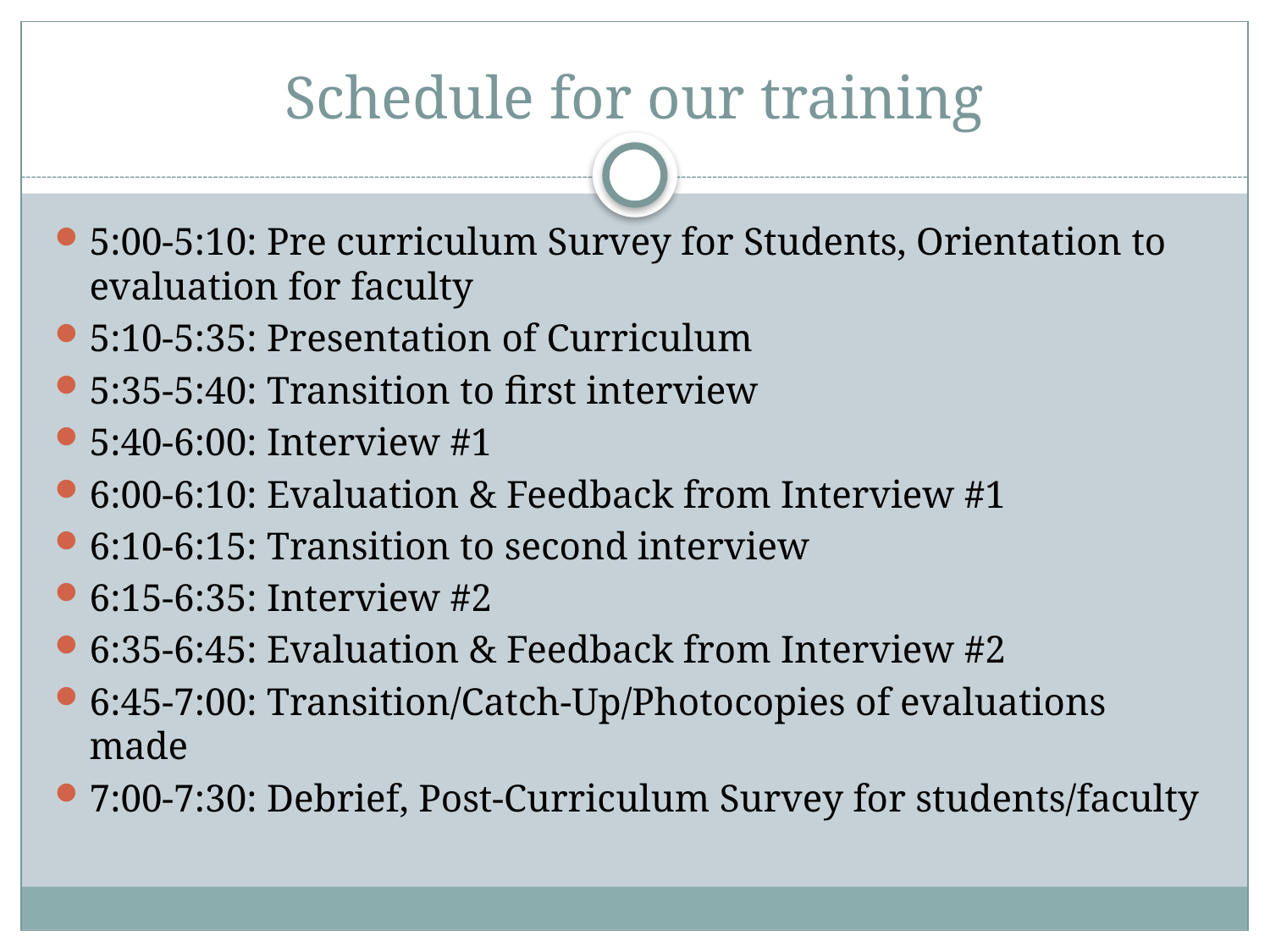

# Schedule for our training
5:00-5:10: Pre curriculum Survey for Students, Orientation to evaluation for faculty
5:10-5:35: Presentation of Curriculum
5:35-5:40: Transition to first interview
5:40-6:00: Interview #1
6:00-6:10: Evaluation & Feedback from Interview #1
6:10-6:15: Transition to second interview
6:15-6:35: Interview #2
6:35-6:45: Evaluation & Feedback from Interview #2
6:45-7:00: Transition/Catch-Up/Photocopies of evaluations made
7:00-7:30: Debrief, Post-Curriculum Survey for students/faculty

## Slide 4
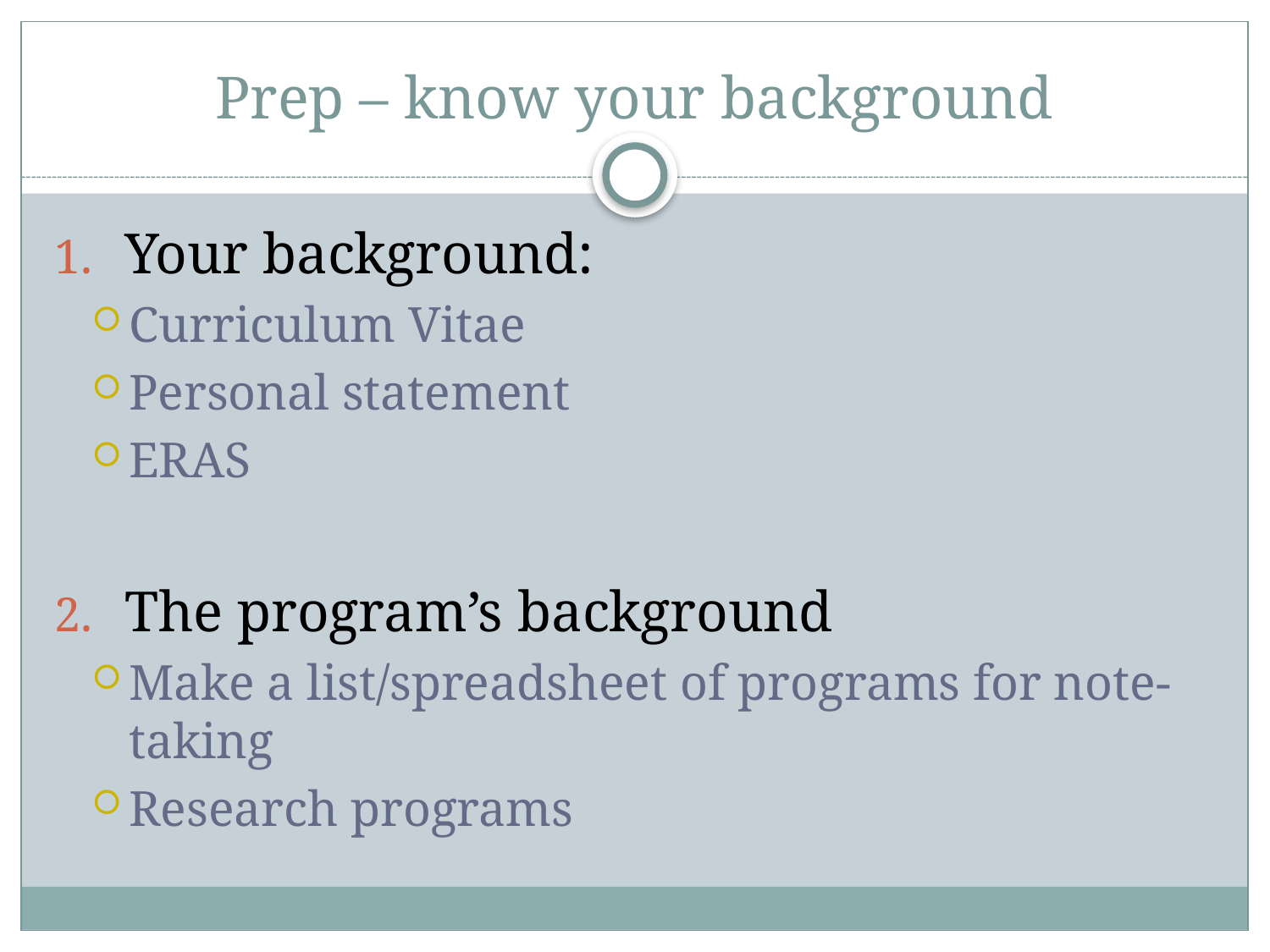

# Prep – know your background
Your background:
Curriculum Vitae
Personal statement
ERAS
The program’s background
Make a list/spreadsheet of programs for note-taking
Research programs

## Slide 5
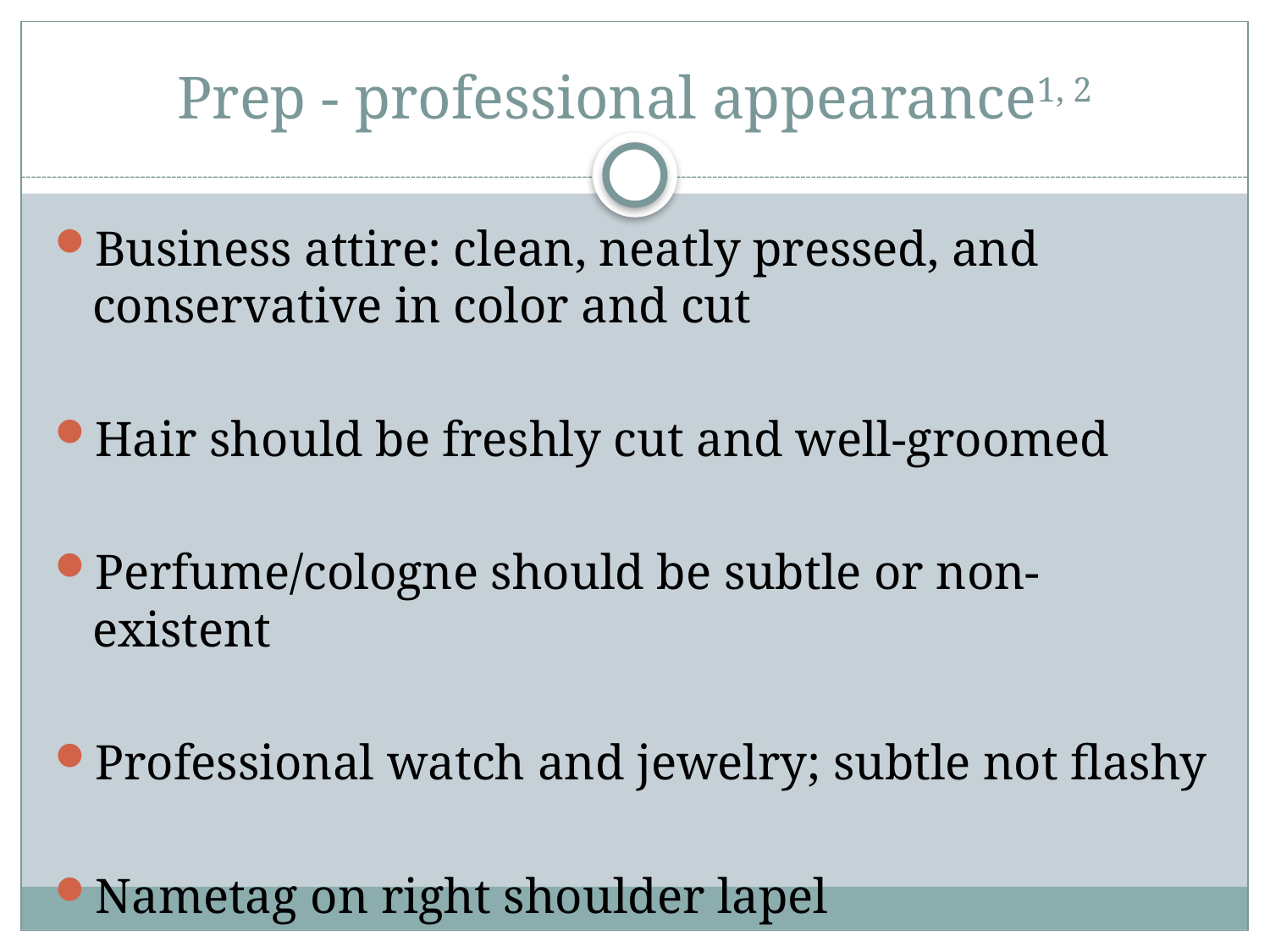

# Prep - professional appearance1, 2
Business attire: clean, neatly pressed, and conservative in color and cut
Hair should be freshly cut and well-groomed
Perfume/cologne should be subtle or non-existent
Professional watch and jewelry; subtle not flashy
Nametag on right shoulder lapel

## Slide 6
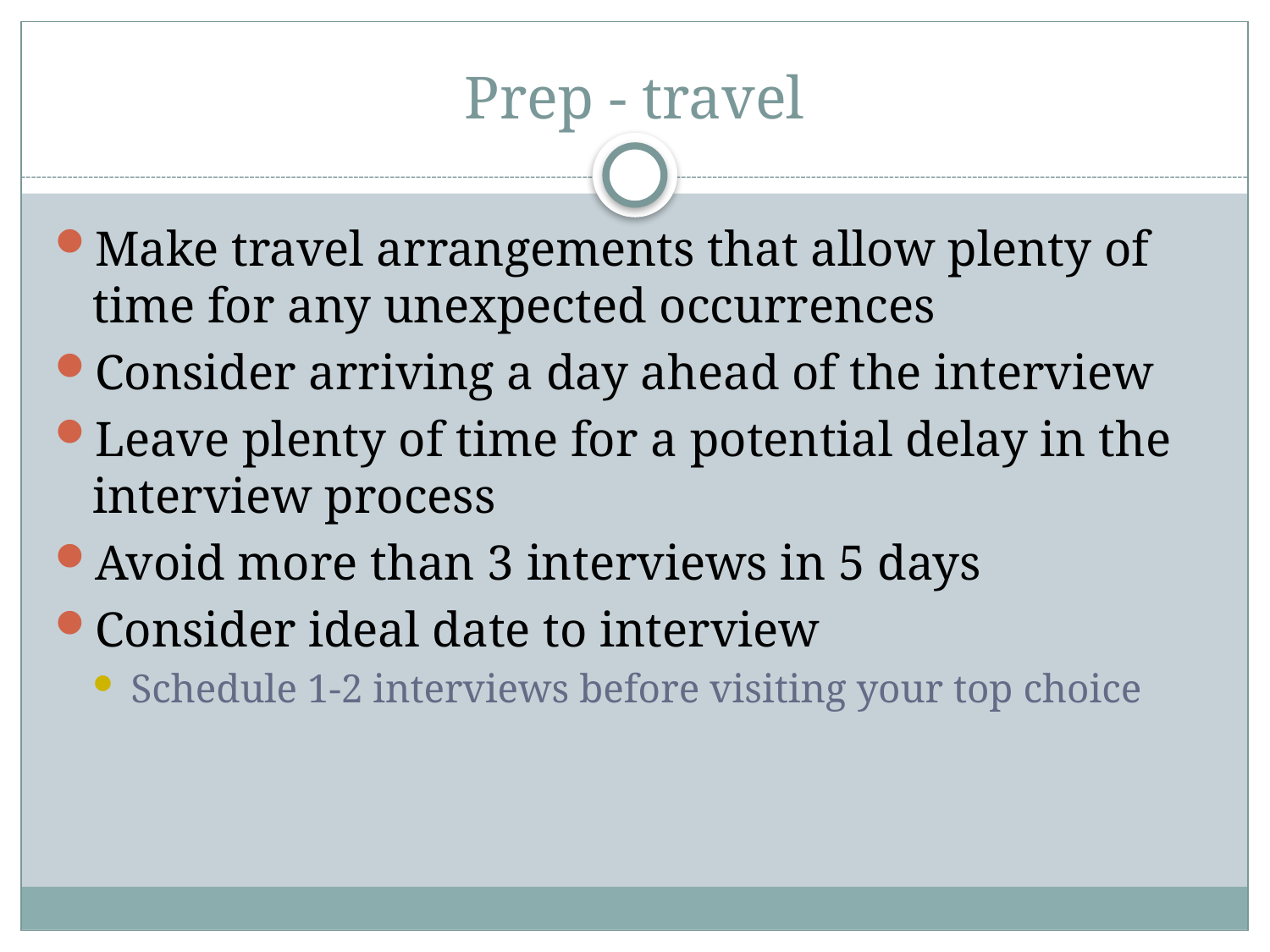

# Prep - travel
Make travel arrangements that allow plenty of time for any unexpected occurrences
Consider arriving a day ahead of the interview
Leave plenty of time for a potential delay in the
interview process
Avoid more than 3 interviews in 5 days
Consider ideal date to interview
Schedule 1-2 interviews before visiting your top choice

## Slide 7
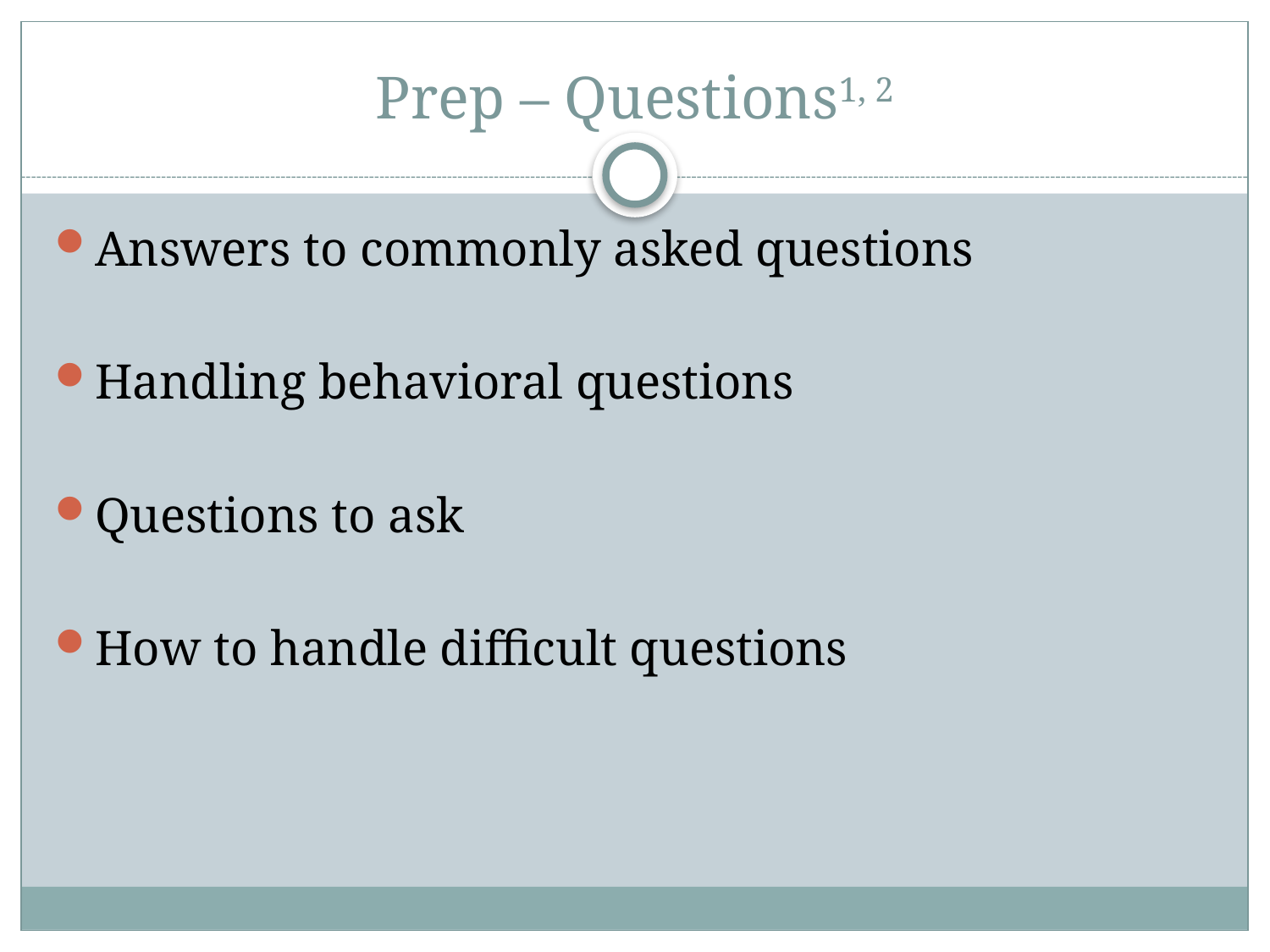

# Prep – Questions1, 2
Answers to commonly asked questions
Handling behavioral questions
Questions to ask
How to handle difficult questions

## Slide 8
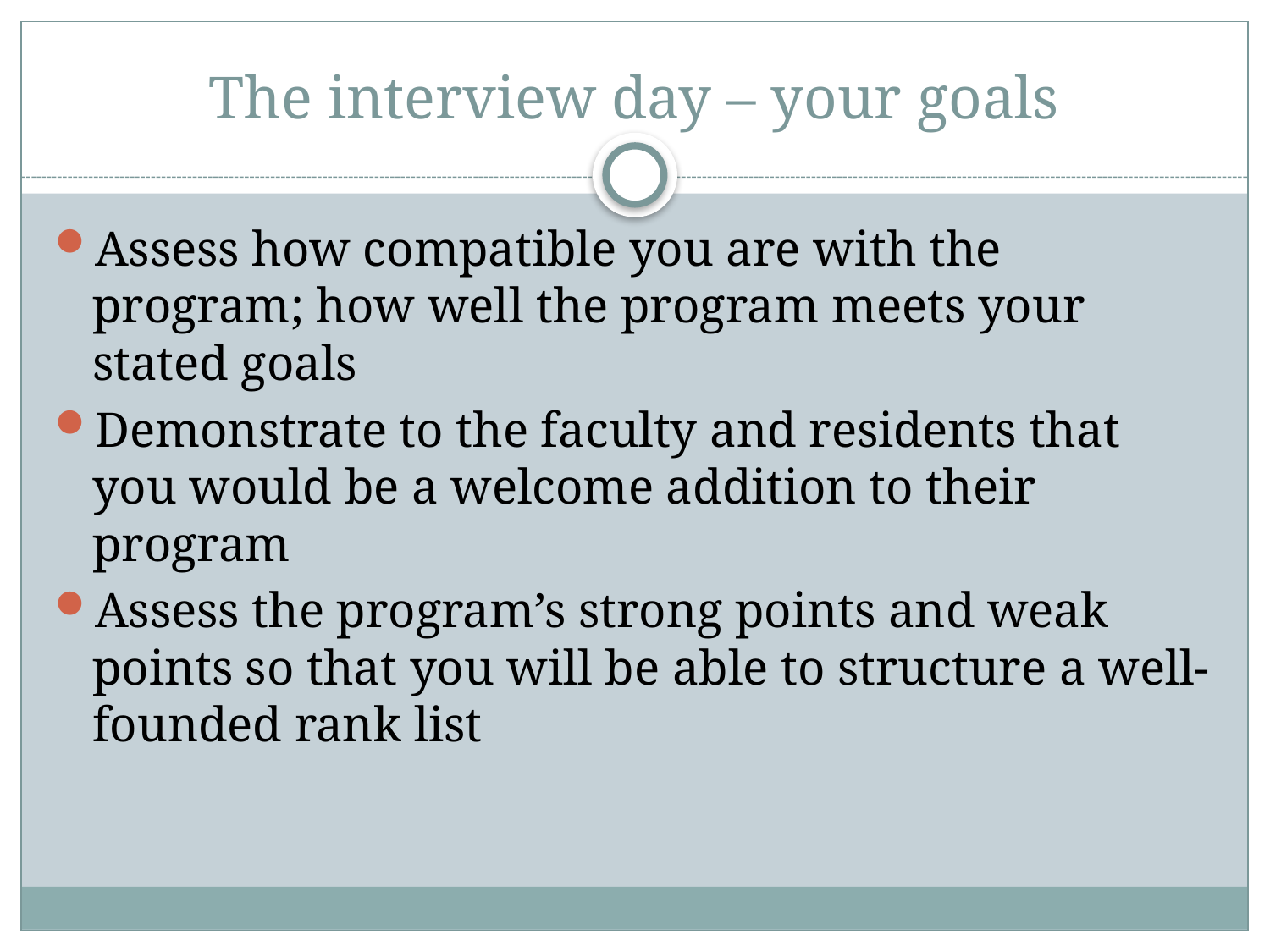

# The interview day – your goals
Assess how compatible you are with the program; how well the program meets your stated goals
Demonstrate to the faculty and residents that you would be a welcome addition to their program
Assess the program’s strong points and weak points so that you will be able to structure a well-founded rank list

## Slide 9
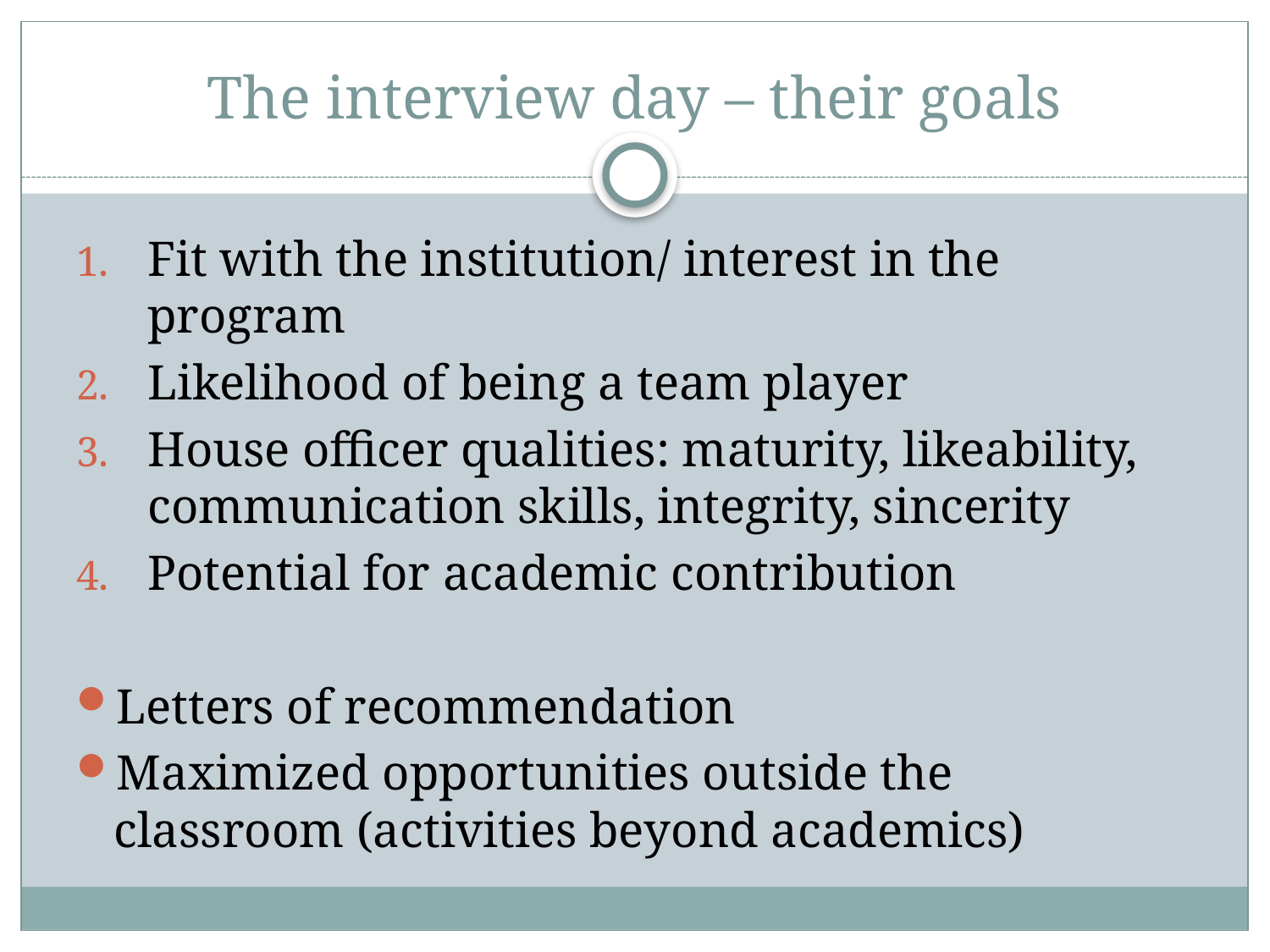

# The interview day – their goals
Fit with the institution/ interest in the program
Likelihood of being a team player
House officer qualities: maturity, likeability, communication skills, integrity, sincerity
Potential for academic contribution
Letters of recommendation
Maximized opportunities outside the classroom (activities beyond academics)

## Slide 10
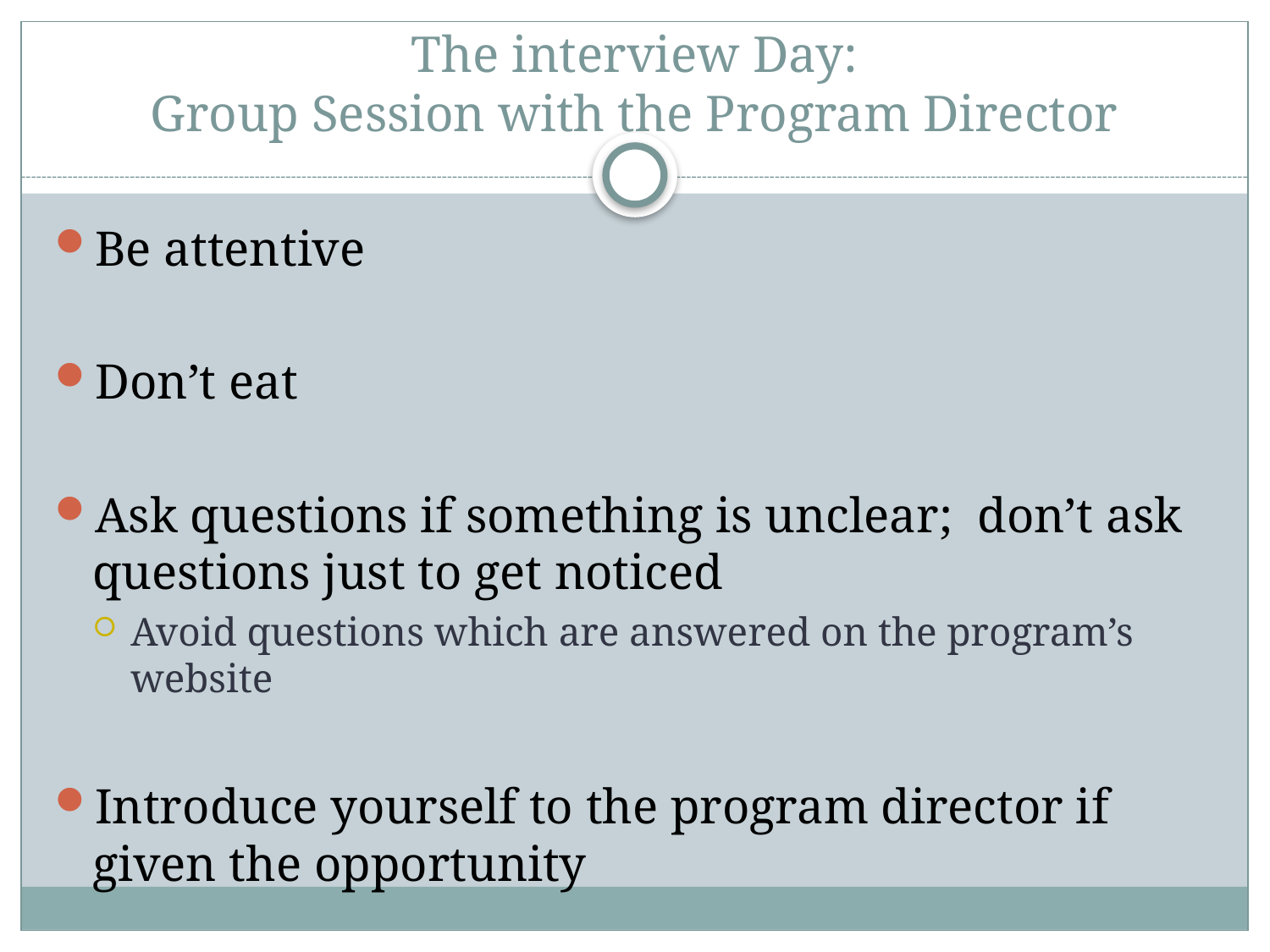

# The interview Day: Group Session with the Program Director
Be attentive
Don’t eat
Ask questions if something is unclear; don’t ask questions just to get noticed
Avoid questions which are answered on the program’s website
Introduce yourself to the program director if given the opportunity

## Slide 11
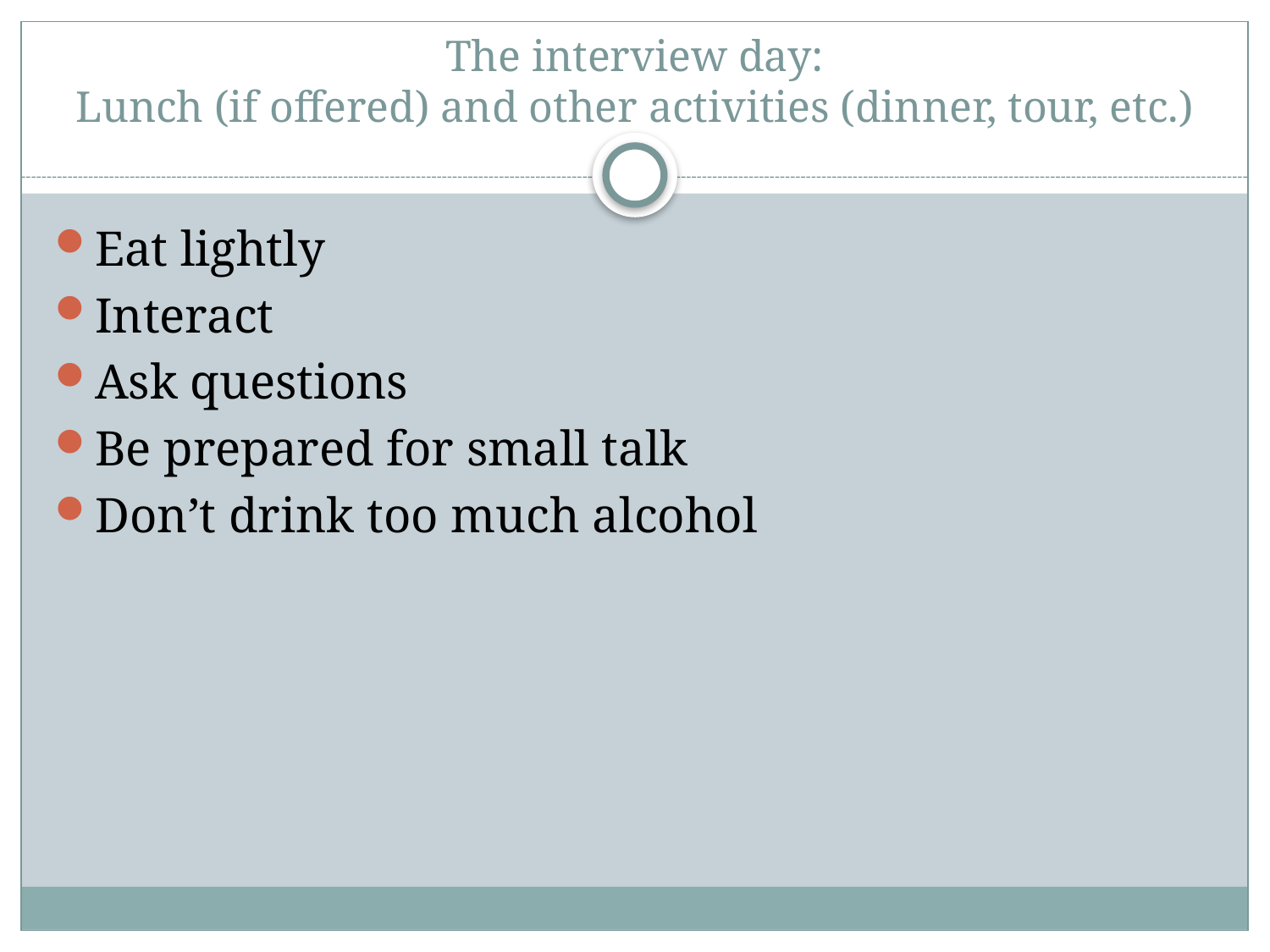

# The interview day: Lunch (if offered) and other activities (dinner, tour, etc.)
Eat lightly
Interact
Ask questions
Be prepared for small talk
Don’t drink too much alcohol

## Slide 12
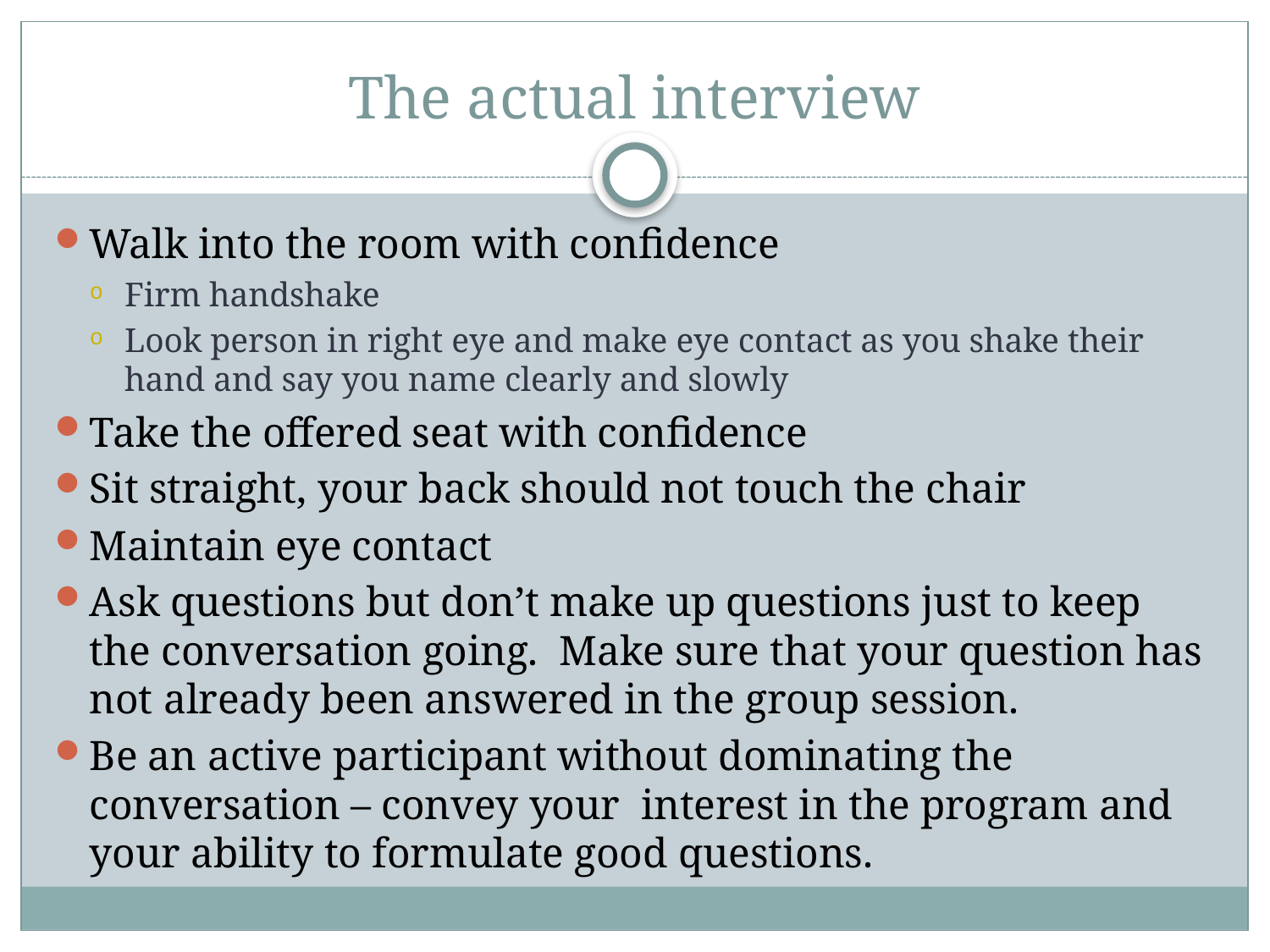

# The actual interview
Walk into the room with confidence
Firm handshake
Look person in right eye and make eye contact as you shake their hand and say you name clearly and slowly
Take the offered seat with confidence
Sit straight, your back should not touch the chair
Maintain eye contact
Ask questions but don’t make up questions just to keep the conversation going. Make sure that your question has not already been answered in the group session.
Be an active participant without dominating the conversation – convey your interest in the program and your ability to formulate good questions.

## Slide 13
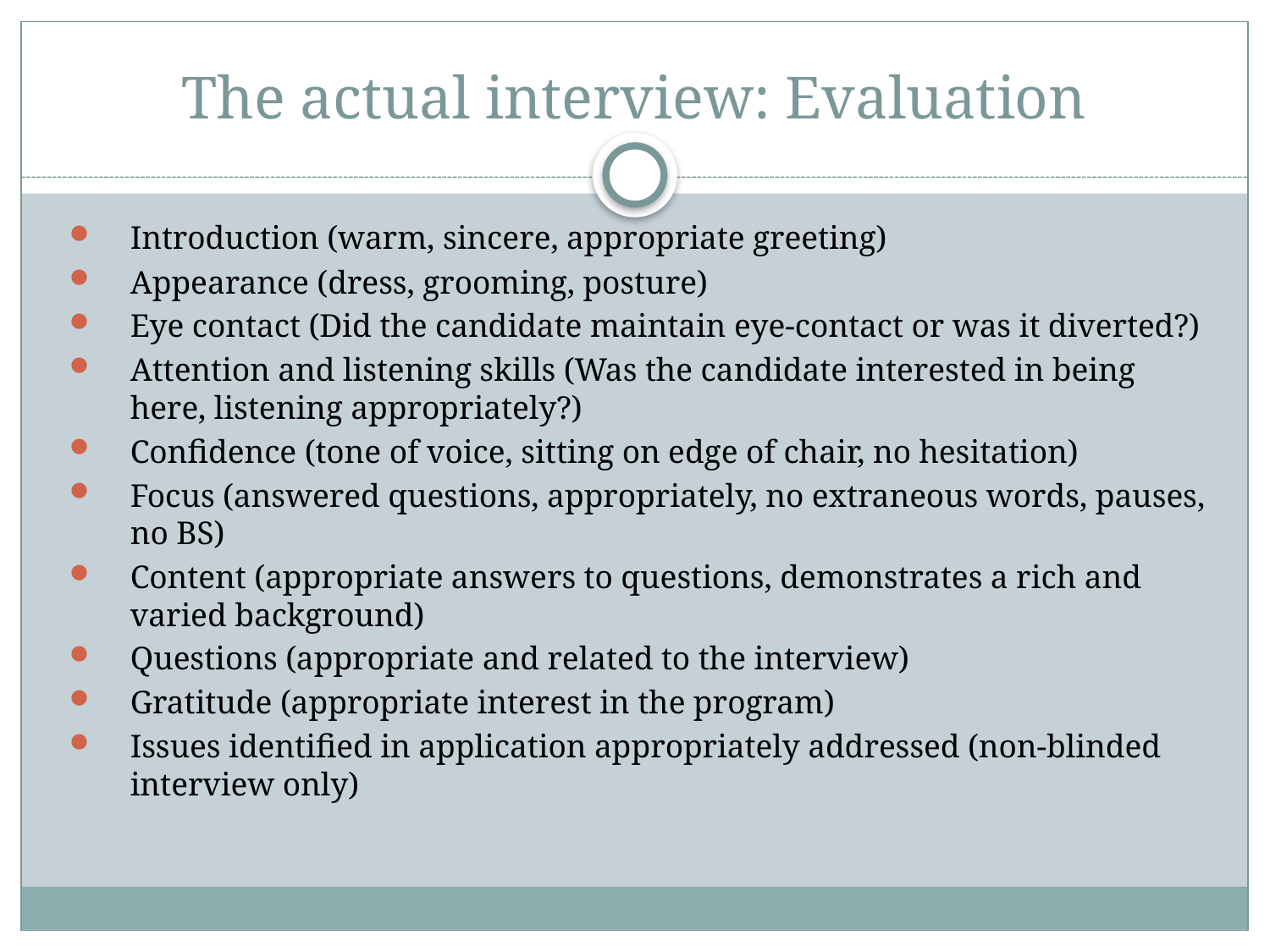

# The actual interview: Evaluation
Introduction (warm, sincere, appropriate greeting)
Appearance (dress, grooming, posture)
Eye contact (Did the candidate maintain eye-contact or was it diverted?)
Attention and listening skills (Was the candidate interested in being here, listening appropriately?)
Confidence (tone of voice, sitting on edge of chair, no hesitation)
Focus (answered questions, appropriately, no extraneous words, pauses, no BS)
Content (appropriate answers to questions, demonstrates a rich and varied background)
Questions (appropriate and related to the interview)
Gratitude (appropriate interest in the program)
Issues identified in application appropriately addressed (non-blinded interview only)

## Slide 14
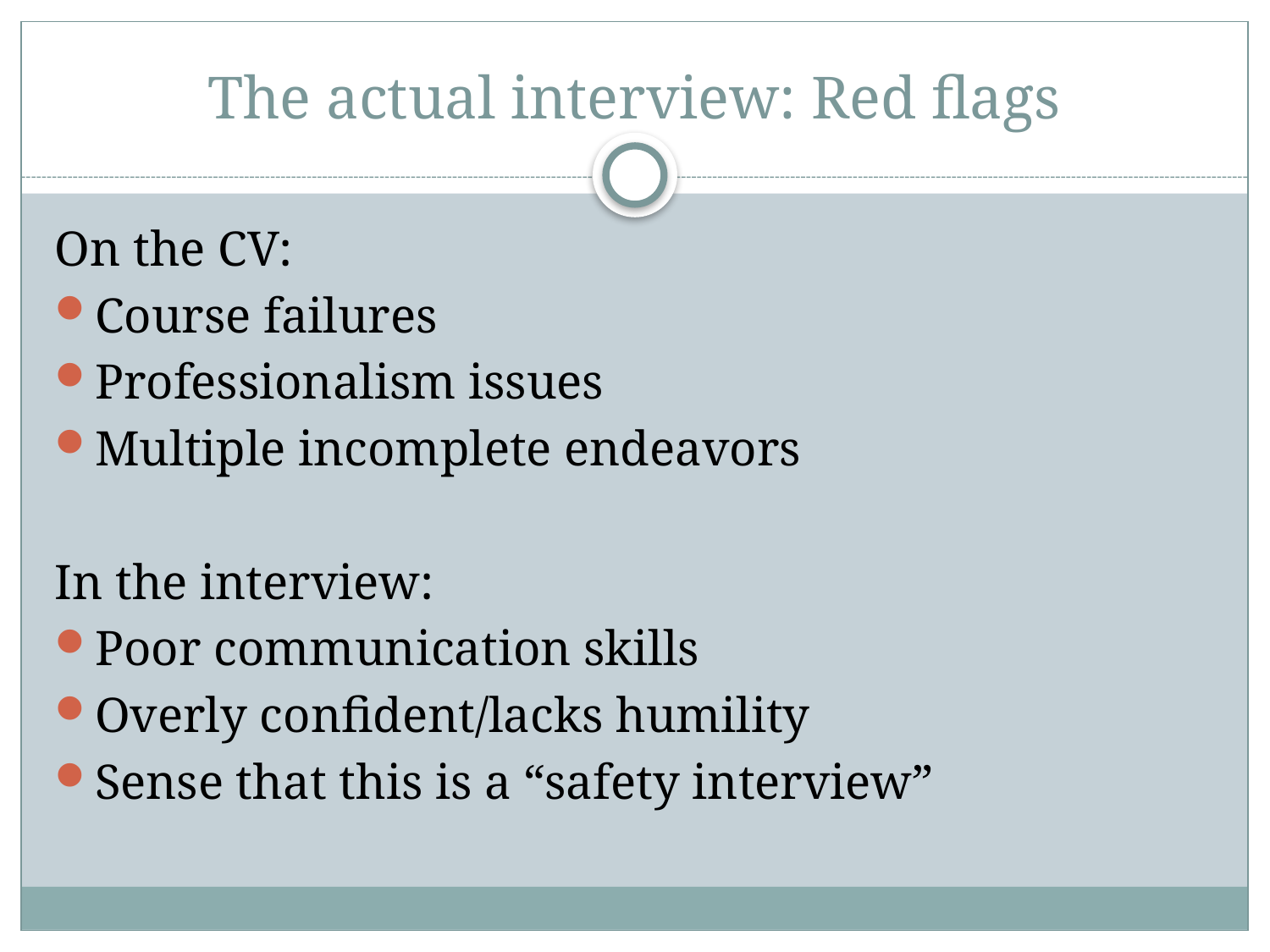

# The actual interview: Red flags
On the CV:
Course failures
Professionalism issues
Multiple incomplete endeavors
In the interview:
Poor communication skills
Overly confident/lacks humility
Sense that this is a “safety interview”

## Slide 15
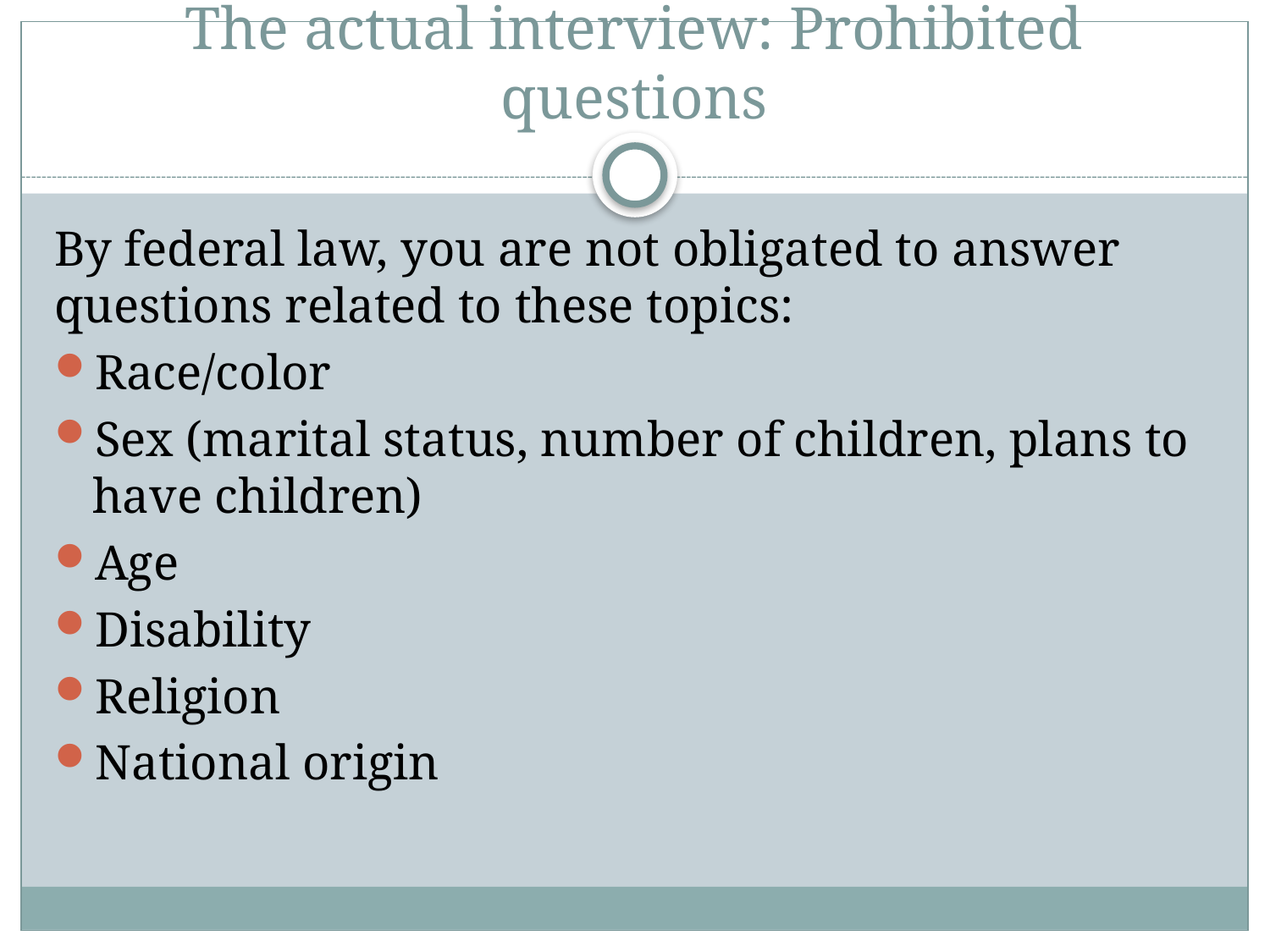

# The actual interview: Prohibited questions
By federal law, you are not obligated to answer questions related to these topics:
Race/color
Sex (marital status, number of children, plans to have children)
Age
Disability
Religion
National origin

## Slide 16
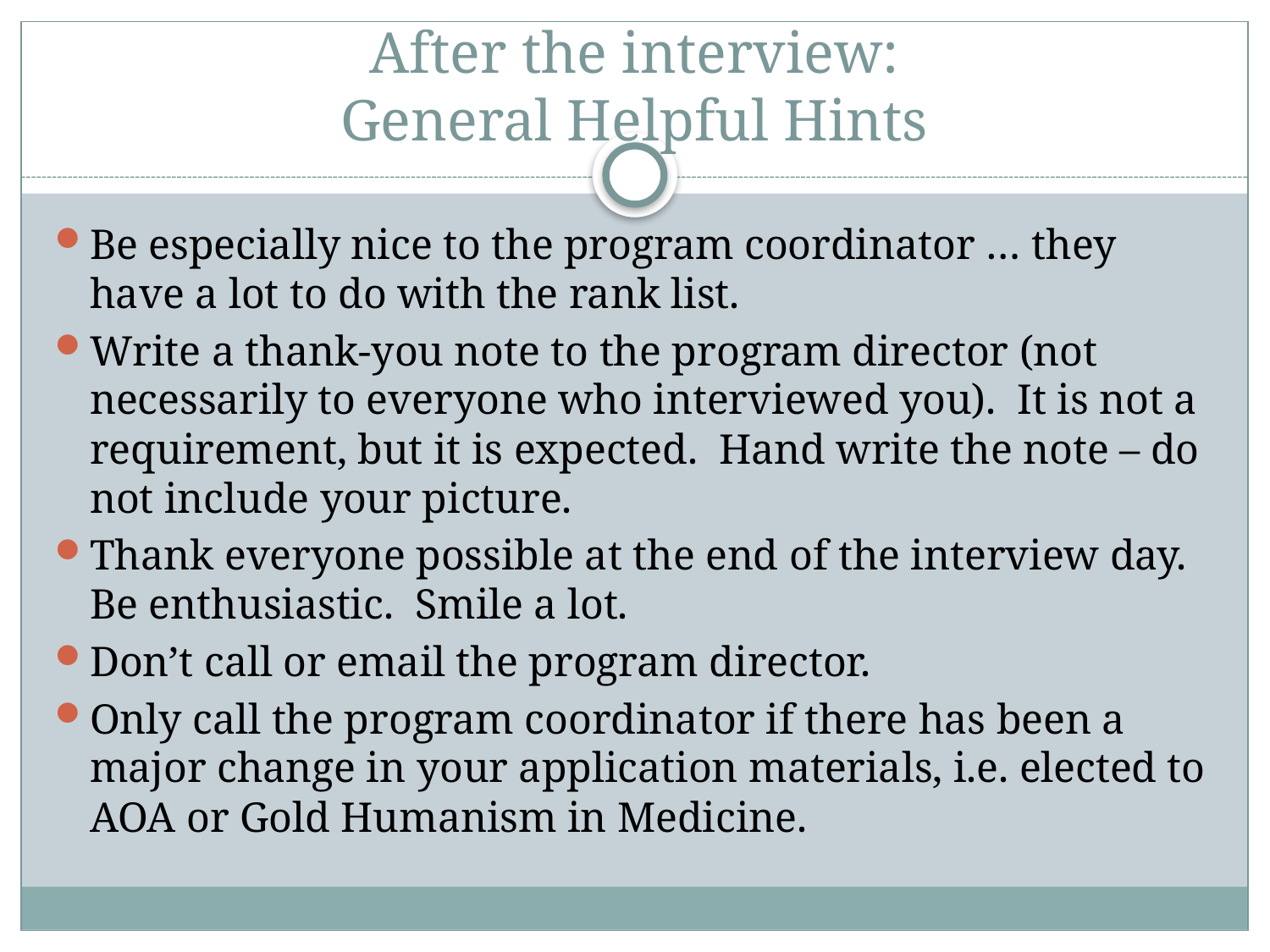

# After the interview: General Helpful Hints
Be especially nice to the program coordinator … they have a lot to do with the rank list.
Write a thank-you note to the program director (not necessarily to everyone who interviewed you). It is not a requirement, but it is expected. Hand write the note – do not include your picture.
Thank everyone possible at the end of the interview day. Be enthusiastic. Smile a lot.
Don’t call or email the program director.
Only call the program coordinator if there has been a major change in your application materials, i.e. elected to AOA or Gold Humanism in Medicine.

## Slide 17
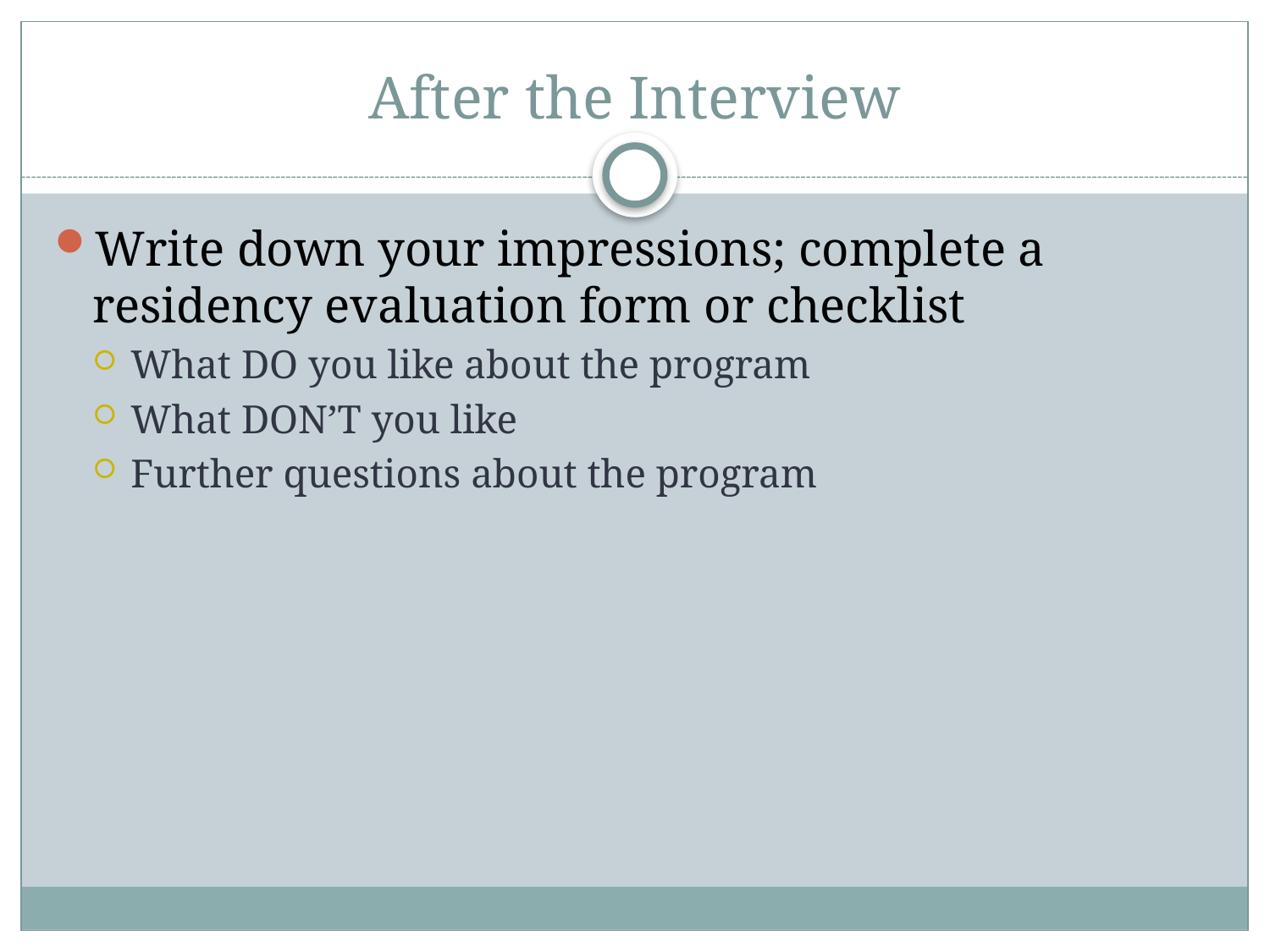

# After the Interview
Write down your impressions; complete a residency evaluation form or checklist
What DO you like about the program
What DON’T you like
Further questions about the program

## Slide 18
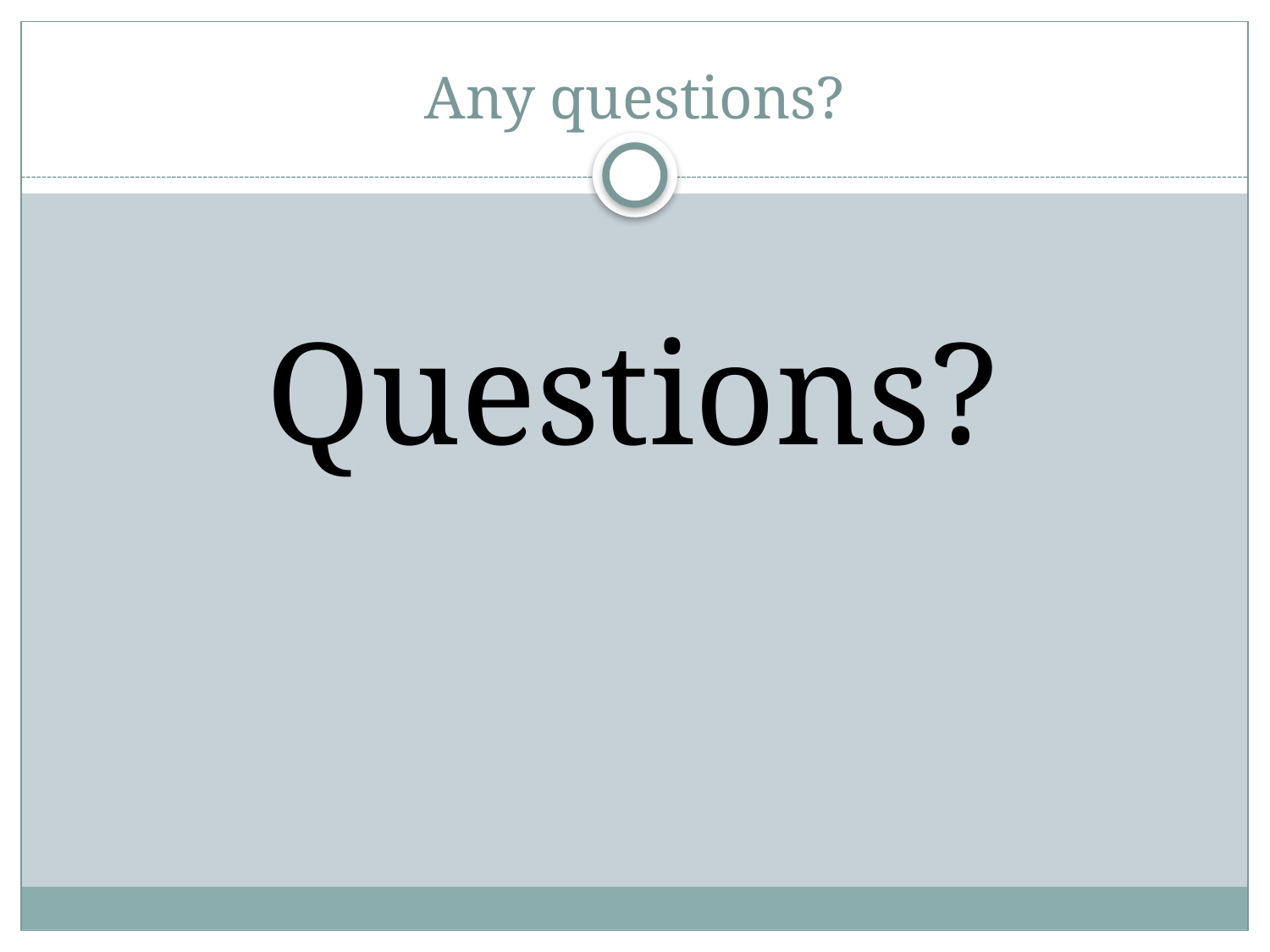

# Any questions?
Questions?

## Slide 19
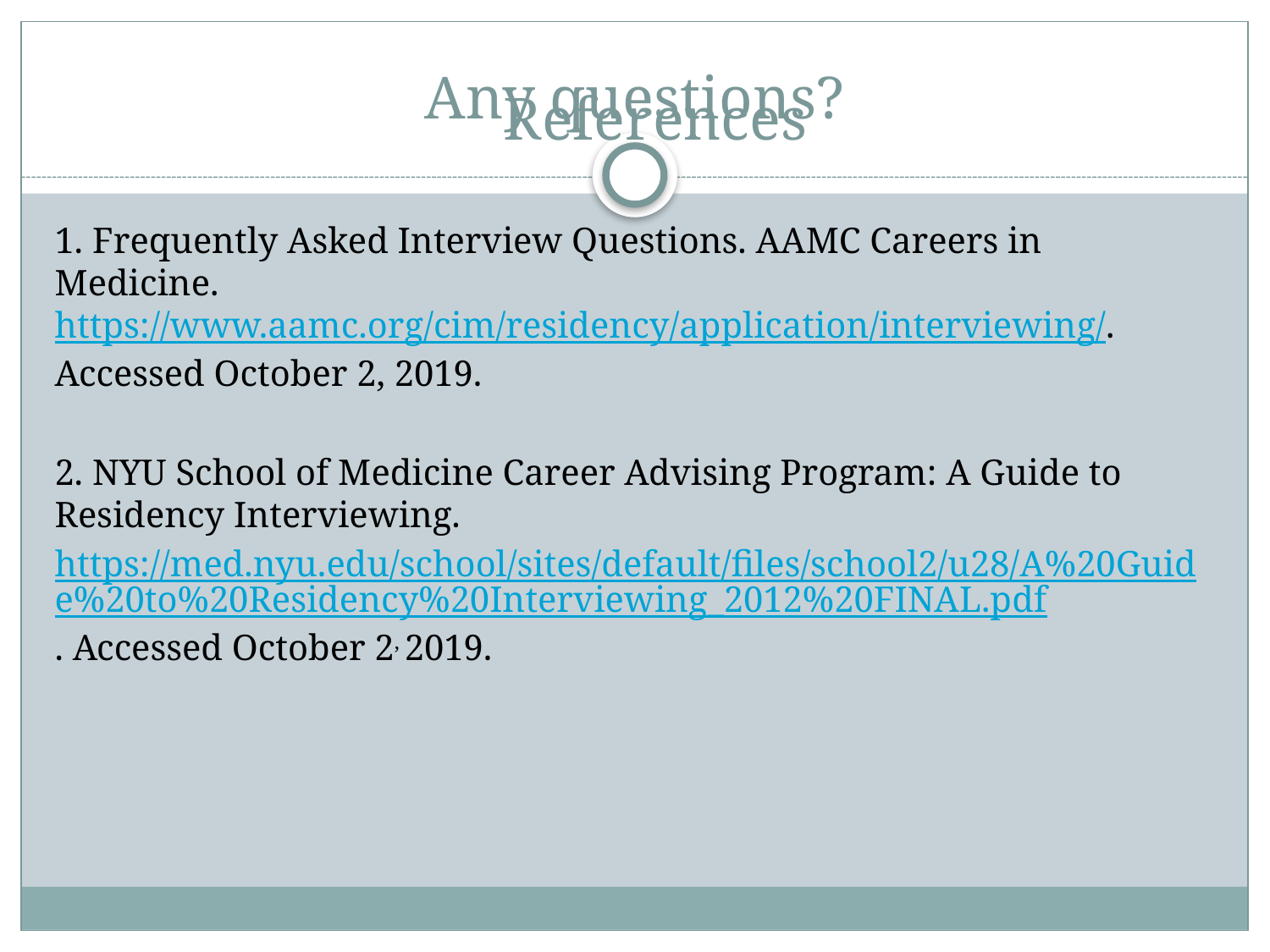

# Any questions?
References
1. Frequently Asked Interview Questions. AAMC Careers in Medicine. https://www.aamc.org/cim/residency/application/interviewing/. Accessed October 2, 2019.
2. NYU School of Medicine Career Advising Program: A Guide to Residency Interviewing.
https://med.nyu.edu/school/sites/default/files/school2/u28/A%20Guide%20to%20Residency%20Interviewing_2012%20FINAL.pdf. Accessed October 2, 2019.
