# Supplementary material for: A Residency Interview Training Program to Improve Medical Student Confidence in the Residency Interview
Source: MedEdPORTAL. 2020 Jul 2;16:10917. doi: 10.15766/mep_2374-8265.10917 (PMC7373200; doi:10.15766/mep_2374-8265.10917)
Supplement: Supplementary file 1 — Didactic Slide Presentation.pptxInformational Packet for Students.docxQuestions for Facilitators.docxInterview Performance Evaluation Tool.docxDebriefing Script.docxGuided Self-Assessment.docxPre- and Posttraining Confidence Survey.docx [file mep_2374-8265.10917-s001.zip › B. Informational Packet for Students.docx]

Interview Training

A Guide to Residency Interviewing

# PRIOR TO THE INTERVIEW:

1. Application
   1. Curriculum Vitae
   2. Personal statement
   3. Electronic Residency Application Service (ERAS)
      1. Including professional head shot.
   4. Letters of Recommendation
   5. Make a list of programs you are interested in
   6. Find out about program from all sources available

- Green Book
- Internet website
- Faculty
- Residents
- Students

# Question Preparation^1, 2^:

C**ommonly asked questions**

- 1. Tell me about yourself?
     1. 15 seconds: brief review of who you are
     2. 30 seconds: educational background
     3. 30 seconds: unique characteristics/strengths
     4. 15 seconds: why you are interested in this program
  2. What are your strengths and weaknesses?
  3. Why should we choose you?
  4. Why are you interested in this specialty?
  5. Why are you interested in this program?
  6. Can you tell me about this deficiency on your record?
  7. Why did you become a doctor?
  8. Tell us about your research experience.
  9. If you could not be a physician, what career would you choose?
  10. What specialty would you pursue if it was not this one?
  11. What leadership roles have you held?
  12. What do you do in your spare time?
      1. What activities do you pursue outside of medicine to maintain balance in your life?
  13. What was your favorite course in medical school?
  14. What are your goals?
  15. Where do you see yourself in 10 years?
  16. Are you interested in academic or clinical medicine?
  17. Do you want to do research?
      1. How would you fund it?
  18. What individual(s) do you consider to have been most influential in your life?
  19. If you could go back in time and meet with one famous person who would it be and why?
  20. What strengths will you bring to a residency program? Conversely, what are the personal weaknesses that you would like to correct?
  21. What are your plans after residency? i.e., private practice, fellowship training, academic medicine, research?
  22. What role did you play in the research project(s) cited in your C.V.? What is your understanding of the purpose and major findings of this research project?
  23. What solutions or ideas would you offer to solve a social problem – aids epidemic, universal health care?
  24. Any question relating to your personal statement, CV, research etc.
  25. Why do you see yourself at our residency program?
      1. Why this program? Why this city?
      2. What are you looking for in a program?
  26. What was most memorable moment of medical school?
  27. Besides our clerkship rotation, what were your favorite rotations? Least favorite rotations?
  28. Describe an ethical dilemma that you noted in medical school and state the pertinent ethical principles.
  29. How would you approach a senior in a conflict, and if you could not resolve the issue internally how would you proceed?
      1. Ethical cases - 1) if you were an intern scrubbed into a surgical case with your attending and he/she started making racial jokes how would you respond? 2) if you were an intern and you felt that your chief resident was not responding appropriately to your concerns about a patient who was SOB with decreased oxygen saturation because your chief wanted to "just hurry up and sign out to the night team" how would you respond? 3) give an example of a time that you made wrong medical assessment of a patient and how you handled that situation.
  30. What do you think of current issues in healthcare?
  31. Explain a challenging situation in your life, and how you handled it.

## Handling Difficult Questions:

Try to anticipate areas of concern and devise plans to overcome them. Do not be surprised if something does not go as planned or if you are posed a difficult question. For example, be prepared to openly discuss a disappointing semester, grade, or having to re- take the USMLE Step 1. Your answers to questions about those experiences should be open and non-defensive.

Tips for Handling Difficult Questions:

- Brainstorm a list of possible questions you may be asked.
- Concentrate on areas that worry you most and solicit help in preparing responses.
- **Think before responding**; there is nothing wrong with pausing briefly first.
- **Be brief and respond in a factual way**. Do not give more information than is necessary.
- Ask the interviewer to restate the question if you do not understand it. Try to determine what information the interviewer seeks.

## Never fabricate or overstate information.

- Anticipate difficult questions, prepare responses in advance, and rehearse.

Not all of the people you interview with will be skilled at conducting interviews.

The law prohibits some types of questions that you are not obligated to answer. Restrictions exist to prevent employers from unfairly eliminating you from consideration. Most illegal questions fall into four broad categories: disabilities and physical skills; personal history; race, ethnicity, or creed; and family and relationship issues. How you handle these types of questions is a personal decision.

First, remember that, in general, these questions are more ignorant than malicious. There is a fine line between questions that are illegal and those that are simply inept, curious, or friendly. Do not approach these questions in a hostile manner.

You should consider carefully whether or not you want to answer. Refusing to answer may reduce your opportunity to make a positive impression. A better option may be to smile, remain pleasant, and answer the question, focusing your comments (for example, for women, on family plans) on the seriousness of your commitment to your training. Some version of “I’m pleased with the job I’ve done thus far balancing my personal and professional life. There shouldn’t be a problem with it in the future” should suffice.

## Don't Forget to Ask: Advice from Residents on

**What to Ask During the Residency Interview**

The process of applying and interviewing for a residency position is complicated and can be stressful. This process involves both “selling” yourself to a program, as well as collecting the information that you will need in deciding how to rank the various programs you visit. Programs that you consider will all have unique strengths and weaknesses—some of which may not immediately apparent. The following list of questions was created by residents and students from various backgrounds as a guide to assist you in identifying and assessing those strengths and weaknesses. Use this guide in constructing your own more specific questions, and in exploring your own expectations and preferences. Your residency training is an important experience. Identifying the program that is best suited to meet your educational and professional expectations is paramount. Some questions are best answered by other residents in the program, and some questions you will need to ask yourself. Ask the program administrators and

residents for specific examples that give a true understanding of the program. Be honest with yourself about how you want your residency experience to be structured. Good luck! And remember, always be yourself.

## Education

- Is there an orientation program for incoming residents?
- Is there a formal didactic curriculum, and what is its structure?
- What are the informal learning opportunities (i.e., bedside rounds, etc.)?
- What programs exist for resident education (e.g. , lectures, journal clubs, grand rounds, board review courses,)?
- Is there a feedback structure that allows for the resident to evaluate the program’s curriculum?
- Is attendance at regional and national conferences encouraged? Is it funded, and, if so, to what degree?
- What are the required rotations for the first year? Subsequent years?
- Are then any required rotations that take place outside of the city?
- Are there opportunities to do “away” rotations?
- Is there a formal mentoring program for new residents, and do faculty serve as mentors?

## Research Opportunities

- Are research opportunities provided to residents? Is this a required experience?
- Is there a possibility of "protected" time for research?
- How are fellowships handled?

## Teaching Responsibilities

- What teaching responsibilities for medical students are expected of residents?
- If residents have teaching responsibilities, how much time per week is spent with students? Is it "protected"?
- Is there any formal training for residents on how to teach students and other learners effectively, and how to provide feedback?

## Clinical Duties

- What is the general call schedule? What provisions are made for back-up call or sick- call coverage?
- What type of structure for supervision is in place?
- Do your residents express that there is an appropriate balance between independence and supervision?
- How does the resident’s autonomy change as he/she progresses through the program?
- What type of ancillary support is available (phlebotomy, respiratory therapy, social workers, etc.)?
- Does the general volume of clinical responsibility support a balance between service and education?
- Do your residents express they are involved in too much non-educational activity (i.e., “scut work”)?

## Resident Performance

- How often are residents evaluated? What is the structure of the evaluation (forms, face-to-face, etc.)?
- What other forms of feedback does the resident receive ( in-training exam, etc.)?
- What support structures are in place for residents in academic need?

## Program Performance

- What is the status of the program’s accreditation?
- If there were any citations at the last review, what has been done to correct them?
- When is the next Residency Review Committee (RRC) review?
- Are there any plans for changing the program size or structure?
- What is the status of the last Accreditation Council for Graduate Medical Education (ACGME) Institutional Review?
- How solid is the financial status of the sponsoring institution?
- How committed is your institution to resident education and graduate medical education in general? How is this evidenced?
- What percent of your residents complete your program?
- What percent of your graduates pass the specialty boards on their first attempt?
- Where do your graduates go (e.g., fellowship, academics, private practice)?

## Employment Issues

- What are the basic resident benefits?
- Is parking a concern for residents at your program?
- Are meals paid for when on call?
- What is your family leave policy?
- Is there reimbursement for educational supplies and books?
- Are moonlighting opportunities available? What are the rules for moonlighting?
- How are residents represented at the institution level? How is the resident member of GMEC selected?
- Is there a union? Is membership mandatory? Are there dues?
- Is there a House Officers Association?

## Questions to specifically ask other Residents

- What are the strengths and weaknesses of the program?
- Would you consider the same program if applying again?
- Is there an appropriate balance between service obligations and the educational program?
- Is there enough ancillary support to minimize "scut?"
- What has changed since you came to the program?
- Is the program responsive to suggestions for change?
- How accessible is the faculty?
- Is the relationship with faculty collegial?
- Do the residents get along with one another?
- How do your residents get along with residents in other programs?
- In what activities are you involved outside of the program?
- How does your spouse/significant other like the city/area?

## Questions to ask Yourself

Finally, you will likely find yourself facing a decision between one of several programs which are all extremely similar from academic and patient-care standpoints. At this time, it is very important to consider factors relating to your personal happiness and comfort for the duration of your residency.

- Can I be happy working in this program and with these people?
- Am I confident in the program and the sponsoring institution?
- Are there factors that make this place (city/town/rural area) an attractive place for me to live during my residency? (Factors that you may include are proximity to immediate and extended family, happiness of spouse/significant other, housing, cost of living, quality of secondary school system, community opportunities, and recreational activities.)

# Logistics:

**Professional Image**

- Clothing should be clean, neatly pressed, and conservative in color and cut
- Scent should be subtle, not overwhelming
- Hair should be freshly cut and well-groomed
- Consider jewelry that is subtle and not flashy

**Travel Arrangements**

- Make travel arrangements that allow plenty of time for any unexpected occurrences. Consider arriving a day ahead of the interview
  - Travel delays can occur from weather, transportation delays, etc.
  - If you fly, always consider a baggage delay
- Leave plenty of time for a potential delay in the interview process.
  - Don’t schedule departure plans too close to the end of the interview session
- Consider the day you might want to interview
  - Try to make sure it is “interview day”
  - Too early? Too late?
- Arrive to the interview session at least ten minutes early

#

# THE INTERVIEW

Group orientation session by program director

- Introduce yourself to the program director if given the opportunity
- Be attentive
- Don’t eat a donut while the presentation is being given
- Ask questions if something is unclear. Don’t ask questions just to get noticed.

Lunch (if offered) and other activities (dinner, tour, etc.)

- Be prepared not to eat much
- Interact with anybody around you
- Ask questions of the residents, faculty
- Be prepared for small talk … brush up on your sports knowledge, current events, movies, books, etc.
- If you go out to a restaurant, don’t drink too much alcohol

# THE EVALUATION

- 1. Medical school record (grades/honors, boards), Dean’s letter
  2. Letter of recommendations (Fair-excellent)
  3. Maximized opportunities outside the classroom (no activities, average level beyond academics, substantial time to interests beyond academics, clearly beyond expectations, high level of creativity and dedication (i.e. multiple publications))
  4. Likelihood of being a team player
  5. House officer qualities: maturity, likeability, communication skills, integrity, sincerity
  6. Potential for academic contribution
  7. Fit with the institution/ interest in our program

## The interview

1. Introduction: Warm, sincere, appropriate greeting
2. Appearance (dress, grooming, posture)
3. Eye contact (did the candidate maintain eye-contact or was it diverted
4. Attention and listening skills (was the candidate interested in being here, listening appropriately
5. Confidence (tone of voice, sitting on edge of chair, no hesitation)
6. Focus (Answered questions, appropriately, no extraneous words, pauses, no BS)
7. Content (Appropriate answers to questions, demonstrates a rich and varied background)
8. Questions (appropriate and related to the interview)
9. Gratitude (appropriate interest in the program)
10. Issues identified in application appropriately addressed (non-blinded interview only

Interviewer Evaluation Tool

Applicant Name:

Interviewer:

| 1 | 2 | 3 | 4 | 0 |
| --- | --- | --- | --- | --- |
| **Introduction** | | | | |
| Does not introduce self, no handshake, no eye contact | Weak hand shake, takes any seat, slouches | Average handshake, good eye contact | Warm, sincere greeting, strong handshake | Dismissive, condescending |
| **Appearance** | | | | |
| Old clothing, short untucked, unkempt hair | Wrinkled outfit, old/scuffed shoes | Average grooming and posture, professional outfit | Well groomed, fresh, pressed, clean professional outfit | Flashy clothing, large jewelry, aggressive posture |
| **Attitude** | | | | |
| Slovenly, disinterested | Quiet, reserved, poor eye contact | Interested, attentive, good eye contact, seems genuine | Actively engaged, upbeat, positive attitude, clearly genuine | Overly enthusiastic, arrogant, haughty |
| **Motivation** | | | | |
| Limited other options | Interested in specialty only because of family expectation or tradition | Longstanding interest in specialty, comments are altruistic, state desire to help others | Has demonstrated personal sacrifice and altruism well beyond expectations | Financially or lifestyle driven |
| **Fit for the Program** | | | | |
| Has no connection or hobbies related to area, has not researched specifics of this program | Moderately interested in program but no connection to area | Interested in program with some connection to area | Connections to the area, knowledgeable of program, asks questions specific to this program | Blatantly disinterested in this program, considers to be a safety interview |
| **Professional Integrity** | | | | |
| Unable to give example | Uses example of others as a role model | Demonstrates personal examples from own experience | Recognizes and describes challenges from own and others’ experiences | Always feels justified or correct, judgmental |
| **Interpersonal Relationships** | | | | |
| Unable to interact well, rude | Limited family contact, relationships limited to others in medicine | Well-rounded relationships, may be uneasy in new situations, | Interacts with new teams easily, networks with others well, | Overachiever, pushy, egocentric, dominates conversations |
| **Challenges/Problem-solving** | | | | |
| Avoids or unable to identify issue, uncomfortable with question | Identifies issue and attempts to answers, halting response | Clearly recognizes problem, cogently delivers organized option assessment | Unusual insight, answers confidently after careful reflection | Resents being asked a scenario, inappropriate |
| **Strengths** | | | | |
| Strengths in 1 of the following areas: education, clinical arena, research, leadership & advocacy | Strengths in 2 of the following areas: education, clinical arena, research, leadership & advocacy | Strengths in 3 of the following areas: education, clinical arena, research, leadership & advocacy | Strengths in all 4 of the following areas: education, clinical arena, research, leadership & advocacy | Believes self to be superior in all domains |
| **Weaknesses** | | | | |
| Unable to identify/lacks insight into any weaknesses | Limited insight into weaknesses and no plan for reconciliation | Insight into weaknesses, superficial plan for how to amend them | Insight into own weaknesses and plan for addressing/ reconciling | Glaring weaknesses, unacceptable to match |
| **Questions** | | | | |
| Has no questions | Asks questions already answered in the interview day’s material | Appropriate and related to the interview | Show extensive background research into the program | Inappropriate or forbidden questions |
| **Summary** | | | | |
| Less than average, hire as last resort,  Rank above 40 | Average candidate,  acceptable,  Top 40 | Above average,  like this candidate,  Top 20 | Excellent, excited to train,  Top 5 | Unacceptable, disruptive,  Do not rank |

Another example of a rating scale: see interview evaluation tool for another example of a rating scale

5 -Outstanding (equivalent to the two best residents in our program) 4-Good (equivalent to the best resident in each year of training)

3-OK, average (equivalent to most of the residents in the program who will finish without any problems)

1. Some problems (equivalent to the resident in each class who is having some problems) 1-Very problematic (equivalent to the resident or two that may be at risk for staying in the program)

Red Flags:

- - Multiple incomplete endeavors
  - Poor communication skills
  - Overly confident/lacks humility
  - Sense that this is a “safety interview”

Post interview etiquette:

- - Be especially nice to the program coordinator … she or he has a lot to do with the rank list
  - Write a thank-you note to the program director (not necessarily to everyone who interviewed you). It is not a requirement, but it is expected. Hand write the note

– do not include your picture.

- - Thank everyone possible at the end of the interview day. Be enthusiastic. Smile a lot.
  - Don’t call the program director, don’t e-mail the program director
  - Only call the program coordinator if there has been a major change in your application materials, i.e. Elected to AOA.

# References

1. Frequently Asked Interview Questions. AAMC Careers in Medicine. <https://www.aamc.org/cim/residency/application/interviewing/>. Accessed October 2, 2019.

2. NYU School of Medicine Career Advising Program: A Guide to Residency Interviewing.

<https://med.nyu.edu/school/sites/default/files/school2/u28/A%20Guide%20to%20Residency%20Interviewing_2012%20FINAL.pdf>. Accessed October 2, 2019.
