# Supplementary material for: A Residency Interview Training Program to Improve Medical Student Confidence in the Residency Interview
Source: MedEdPORTAL. 2020 Jul 2;16:10917. doi: 10.15766/mep_2374-8265.10917 (PMC7373200; doi:10.15766/mep_2374-8265.10917)
Supplement: Supplementary file 1 — Didactic Slide Presentation.pptxInformational Packet for Students.docxQuestions for Facilitators.docxInterview Performance Evaluation Tool.docxDebriefing Script.docxGuided Self-Assessment.docxPre- and Posttraining Confidence Survey.docx [file mep_2374-8265.10917-s001.zip › C. Questions for Facilitators.docx]

Interview Simulation Training Questions for Facilitators

1. Tell me about yourself?
2. What are your strengths?
3. What are your weaknesses?
4. Why this specialty?
5. What are your plans for after residency?
6. What are you looking for in a program?
7. Why our program?
8. Tell me about your research experience
9. Tell me about a challenging case, or a challenge in your life that you have had to overcome?
