# Supplementary material for: A Residency Interview Training Program to Improve Medical Student Confidence in the Residency Interview
Source: MedEdPORTAL. 2020 Jul 2;16:10917. doi: 10.15766/mep_2374-8265.10917 (PMC7373200; doi:10.15766/mep_2374-8265.10917)
Supplement: Supplementary file 1 — Didactic Slide Presentation.pptxInformational Packet for Students.docxQuestions for Facilitators.docxInterview Performance Evaluation Tool.docxDebriefing Script.docxGuided Self-Assessment.docxPre- and Posttraining Confidence Survey.docx [file mep_2374-8265.10917-s001.zip › D. Interview Performance Evaluation Tool.docx]

Interview Performance Evaluation

Applicant Name:

Interviewer:

| 1 | 2 | 3 | 4 | 0 |
| --- | --- | --- | --- | --- |
| **Introduction** | | | | |
| Does not introduce self, no handshake, no eye contact | Weak hand shake, takes any seat, slouches | Average handshake, good eye contact | Warm, sincere greeting, strong handshake | Dismissive, condescending |
| **Appearance** | | | | |
| Old clothing, short untucked, unkempt hair | Wrinkled outfit, old/scuffed shoes | Average grooming and posture, professional outfit | Well groomed, fresh, pressed, clean professional outfit | Unprofessional  clothing, aggressive posture |
| **Attitude** | | | | |
| Slovenly, disinterested | Quiet, reserved, poor eye contact | Interested, attentive, good eye contact, seems genuine | Actively engaged, upbeat, positive attitude, clearly genuine | Overly enthusiastic, arrogant, haughty |
| **Motivation** | | | | |
| Limited other options | Interested in specialty only because of family expectation or tradition | Longstanding interest in specialty, comments are altruistic, state desire to help others | Has demonstrated personal sacrifice and altruism well beyond expectations | Financially or lifestyle driven |
| **Fit for the Program** | | | | |
| Has no connection or hobbies related to area, has not researched specifics of this program | Moderately interested in program but no connection to area | Interested in program with some connection to area | Connections to the area, knowledgeable of program, asks questions specific to this program | Blatantly disinterested in this program, considers to be a safety interview |
| **Professional Integrity** | | | | |
| Unable to give example | Uses example of others as a role model | Demonstrates personal examples from own experience | Recognizes and describes challenges from own and others’ experiences | Always feels justified or correct, judgmental |
| **Interpersonal Relationships** | | | | |
| Unable to interact well, rude | Limited family contact, relationships limited to others in medicine, | Well-rounded relationships, may be uneasy in new situations, | Interacts with new teams easily, networks with others well, | Overachiever, pushy, egocentric, dominates conversations |
| **Challenges/Problem-solving** | | | | |
| Avoids or unable to identify issue, uncomfortable with question | Identifies issue and attempts to answers, halting response | Clearly recognizes problem, cogently delivers organized option assessment | Unusual insight, answers confidently after careful reflection | Resents being asked a scenario, inappropriate |
| **Strengths** | | | | |
| Strengths in 1 of the following areas: education, clinical arena, research, leadership & advocacy | Strengths in 2 of the following areas: education, clinical arena, research, leadership & advocacy | Strengths in 3 of the following areas: education, clinical arena, research, leadership & advocacy | Strengths in all 4 of the following areas: education, clinical arena, research, leadership & advocacy | Believes self to be superior in all domains |
| **Weaknesses** | | | | |
| Unable to identify/lacks insight into any weaknesses | Limited insight into weaknesses and no plan for reconciliation | Insight into weaknesses, superficial plan for how to amend them | Insight into own weaknesses and plan for addressing/ reconciling | Glaring weaknesses, unacceptable to match |
| **Questions** | | | | |
| Has no questions | Asks questions already answered in the interview day’s material | Appropriate and related to the interview | Show extensive background research into the program | Inappropriate or forbidden questions |
| **Summary** | | | | |
| Less than average, hire as last resort,  Rank below 40 | Average candidate,  acceptable,  Top 40 | Above average,  like this candidate,  Top 20 | Excellent, excited to train,  Top 5 | Unacceptable, disruptive,  Do not rank |
