# Supplementary material for: A Residency Interview Training Program to Improve Medical Student Confidence in the Residency Interview
Source: MedEdPORTAL. 2020 Jul 2;16:10917. doi: 10.15766/mep_2374-8265.10917 (PMC7373200; doi:10.15766/mep_2374-8265.10917)
Supplement: Supplementary file 1 — Didactic Slide Presentation.pptxInformational Packet for Students.docxQuestions for Facilitators.docxInterview Performance Evaluation Tool.docxDebriefing Script.docxGuided Self-Assessment.docxPre- and Posttraining Confidence Survey.docx [file mep_2374-8265.10917-s001.zip › E. Debriefing Script.docx]

**Post-Training, Large Group, Debrief Script**

We use the Promoting Excellence and Reflective Learning in Simulation (PEARLS) debriefing script^1^. This script is specifically designed for new debriefers and can be easily adapted to multiple activities. This script has four domains: reaction, description, analysis, and application.

**Facilitators leading the debriefing session can use the following questions to guide the discussion:**

1. **How is everyone feeling? Or how did that feel?** (Reaction)
2. **Can someone summarize the interview experience?** (Description)
3. **What aspects of the interview went well?** (Analysis)
4. **What aspects of the interview were challenging?** (Analysis)
5. **What is one take-away that will help you in the future? Or what is one thing you will change to perform better in a future interview?** (Application)

Reference

1. Eppich W, Cheng A. Promoting Excellence and Reflective Learning in Simulation (PEARLS): Development and Rationale for a Blended Approach to Health Care Simulation Debriefing. Simulation in Healthcare. 2015;10(2):106-115.
