# Supplementary material for: A Residency Interview Training Program to Improve Medical Student Confidence in the Residency Interview
Source: MedEdPORTAL. 2020 Jul 2;16:10917. doi: 10.15766/mep_2374-8265.10917 (PMC7373200; doi:10.15766/mep_2374-8265.10917)
Supplement: Supplementary file 1 — Didactic Slide Presentation.pptxInformational Packet for Students.docxQuestions for Facilitators.docxInterview Performance Evaluation Tool.docxDebriefing Script.docxGuided Self-Assessment.docxPre- and Posttraining Confidence Survey.docx [file mep_2374-8265.10917-s001.zip › F. Guided Self-Assessment.docx]

**Simulated Interview Video Checklist**

1. Introduction:
   1. Good eye contact No (0) Yes (1)
   2. Firm handshake No (0) Yes (1)
   3. Said name clearly and slowly No (0) Yes (1)
   4. Sincere, professional greeting No (0) Yes (1)
   5. Took chair gracefully, confidently No (0) Yes (1)
2. Appearance:
   1. Hair Unkempt (0) fresh cut, off face, professional (1)
   2. Face/facial hair Distracting makeup, facial hair unkempt (0) Professional (1)
   3. Jewelry/piercings Distracting, large (0) Professional (1)
   4. Top Wrinkled, bad neckline, too bright (0) Pressed, clean professional (1)
   5. Bottom Wrinkled, bad fit, skirt too short (0) Pressed, clean, professional (1)
   6. Socks/shoes Wrinkled, don’t match, scuffed (0) Matching, polished (1)
3. Non-verbal communication
   1. Body Lean Backward (0) Neutral (1) Forward (2)
   2. Body Position Closed (0) Open (1)
   3. Postural Change Yes (0) No (1)
   4. Facial Expression Blank or mismatched (0) Adequately expressive (1)
   5. Eye contact More likely when talking (0) Equal when talking and listening (1)
   6. Affirmative gestures Infrequent (0) Adequately frequent (1)
   7. Non-purposeful movements Frequent (0) Few or none (1)
   8. Hand gestures Frequent (0) Few or none (1)
4. Attitude:
   1. Disinterested (0) Quiet, reserved (1) interested, attentive (2)

engaged, positive, genuine (3)

1. Language:
   1. Uses slang, overuse of word “like” (0) basic vocabulary (1) strong vocabulary, professional language (2)
2. Tone of voice:
   1. Dull (0) Average (1) Bright (2)
3. Speed of speech:
   1. Slow, lots of dead space (0) a little too fast (1) good speed, conversational, pauses are appropriate (2)
4. Content:
   1. Answers are vague, lacking substance, too short (0) Answers are thoughtful, organized, substantive, professional (2)

COMMENTS (WHAT WAS SAID): General comments about the content about what you said. Use specific examples of questions and answers and what you could have said that would have been better.

COMMENTS (HOW IT WAS SAID): General comments about how you said certain responses. Use specific examples of questions and answers and how you could have said it better.
