# Supplementary material for: A Residency Interview Training Program to Improve Medical Student Confidence in the Residency Interview
Source: MedEdPORTAL. 2020 Jul 2;16:10917. doi: 10.15766/mep_2374-8265.10917 (PMC7373200; doi:10.15766/mep_2374-8265.10917)
Supplement: Supplementary file 1 — Didactic Slide Presentation.pptxInformational Packet for Students.docxQuestions for Facilitators.docxInterview Performance Evaluation Tool.docxDebriefing Script.docxGuided Self-Assessment.docxPre- and Posttraining Confidence Survey.docx [file mep_2374-8265.10917-s001.zip › G. Pre- and Posttraining Confidence Survey.docx]

**Pre- and Post-Training Confidence Survey**

**Strongly Disagree Agree**

**1 2 3 4 5**

**Please rate the extent to which you agree or disagree with the following statements:**

**Because of this training:**

1. I have increased my knowledge of the interview process. 1 2 3 4 5
2. I have learned new skills. 1 2 3 4 5
3. I have increased confidence in my ability to interview. 1 2 3 4 5
4. I am better prepared for my interview. 1 2 3 4 5
5. I have accomplished my goals for this training. 1 2 3 4 5

**Strongly Disagree Agree**

1. **2 3 4 5**

**Please rate the extent to which you agree or disagree with the following statements:**

**Because of this training:**

1. I will apply this new knowledge and skills to the authentic residency interviews. 1 2 3 4 5
2. I will improve my interview performance. 1 2 3 4 5
3. I will increase my likelihood of matching in my desired specialty. 1 2 3 4 5

| **Comments:** |
| --- |
